# Neutron Conversion Layers for Semiconductor Neutron Detectors: A Review of Materials, Thin-Film Deposition Technologies, and Performance Optimization

**DOI:** 10.3390/s26216901

**Published:** 2026-10-31

**Authors:** Jiayao Li, Wenming Xia, Junjun Gong, Chengqiang Liang, Lei Zhang, Chunyuan Wang

**Affiliations:** Naval University of Engineering, Wuhan 430033, China; m25185803@nue.edu.cn (J.L.); 0908051018@nue.edu.cn (J.G.); 2120211068@nue.edu.cn (C.L.); m25182705@nue.edu.cn (L.Z.); x24182703@nue.edu.cn (C.W.)

**Keywords:** Semiconductor neutron detectors, neutron conversion layers, boron carbide, lithium fluoride, thin-film deposition, 3D microstructures, microstructured semiconductor neutron detector (MSND)

## Abstract

Semiconductor neutron detectors are compact, low-voltage alternatives to ^3^He-based detectors, and their detection performance hinges on the conversion layer that transforms neutrons into charged particles. Previous reviews have treated this layer as an auxiliary component rather than as a thin-film engineering problem. Here, four representative conversion materials (^6^LiF, ^10^B_4_C, BN, and Gd) are evaluated against five deposition technologies (magnetron sputtering, vacuum evaporation, solution-based deposition, chemical vapor deposition, and atomic layer deposition) within a four-dimensional framework of areal density, density/uniformity, conformality, and adhesion/interface quality. The comparison shows that performance differences among deposition technologies trace to their deposition mechanisms: PVD processes enable large-area planar films but lack three-dimensional conformality; CVD and ALD coat conformally, but the isotope areal densities they deliver are rarely reported and therefore remain unquantified; solution-based methods scale cheaply but lose efficiency to binder dilution and porosity. Three-dimensional microstructured architectures relax the planar self-absorption constraint, with intrinsic efficiencies well above the ~4–5% ceiling of the single-sided single-layer planar configuration, although device-level validation of conformal deposition rests on only a few reports, principally LPCVD ^10^B trench filling, with reported intrinsic efficiencies of 16.3 ± 1.6% and 26.1 ± 0.3%, and interfacial dead layers and radiation damage remain key reliability concerns. Magnetron-sputtered ^10^B_4_C is currently the most mature option; further progress requires cross-process thin-film benchmarking, device-level testing of conformal coating on 3D microstructures, and semiconductor-grade material quality in emerging functional-integration candidates such as h-BN.

## 1. Introduction

### 1.1. Research Background

Neutron detection is fundamental to many fields, including nuclear safety monitoring, high-energy physics experiments, fusion diagnostics, space radiation detection, and homeland security. Conventional neutron detection technologies, such as ^3^He proportional counters, Bonner sphere spectrometers, and track-etch detectors, have served neutron measurement well, but their limitations are increasingly evident. In particular, the global shortage of ^3^He since the early 2000s has stimulated intensive efforts toward developing alternative neutron detection technologies [1]. In this context, semiconductor neutron detectors have emerged as a promising replacement technology for ^3^He detectors due to their compact structure, low operating voltage (typically several volts), rapid response capability, compatibility with semiconductor fabrication processes, and avoidance of high-pressure gas sealing. Consequently, semiconductor neutron detectors have been recognized by organizations in the United States, Europe, and Japan as a priority direction for next-generation neutron detection technologies [2]. The detectors discussed throughout this review are designed for thermal neutrons (E ≈ 25 meV), where the capture cross sections of ^10^B, ^6^Li and Gd reach their largest values and external conversion layers are most competitive; fast neutrons are accessible only after moderation or through recoil-nucleus processes (Section 2.3). The one partial exception is the gadolinium-based conversion layer: owing to its pronounced resonance absorption, Gd retains appreciable capture cross section well beyond the 1/v regime, so Gd-based detectors also respond in the epithermal range (Section 2.3 and Section 3.4).

Because neutrons carry no electric charge, they cannot directly produce ionization signals in semiconductor materials. Instead, semiconductor neutron detectors rely on a conversion process, where neutron interactions with high-cross-section conversion materials generate charged secondary particles, such as α particles, ^7^Li ions, and tritons, which are subsequently detected through charge generation within the semiconductor sensitive region [3,4,5,6]. In semiconductor neutron detectors, the surface-deposited neutron conversion layer plays a central role in transforming neutrons into detectable charged particles. Its material composition, deposition strategy, and microstructural quality fundamentally determine the achievable detection efficiency, n/γ discrimination capability, and long-term device reliability [7]. Therefore, this review specifically focuses on conversion-layer thin-film engineering from a materials and process perspective.

Although this design strategy has been implemented for over two decades, neutron conversion layers have remained an underexplored component in existing review literature. Pietropaolo reviewed the fundamental physical mechanisms of neutron detection, including nuclear reaction pathways and sensor operating principles, but the preparation technologies of neutron conversion layers were beyond the scope of that study [8]. Cheng et al. summarized ultra-wide-bandgap semiconductor radiation detectors; however, neutron conversion layers were only briefly introduced as a structural element within the overall device configuration rather than being analyzed as an independent functional component [4]. Wang et al. reviewed high-temperature radiation-tolerant neutron detectors in 2025, but their discussion of conversion layers was primarily limited to candidate material selection [5]. Kosinov et al. employed CdTe thin-film detectors for coupled simulations of ionizing radiation and thermal neutron detection, demonstrating the feasibility of integrating neutron conversion layers with thin semiconductor detector platforms [9]. Angelone and Verona reviewed diamond-based neutron detectors in 2021, while the processing strategies and engineering considerations of neutron conversion layers remained insufficiently discussed [6]. Ruddy et al. reviewed silicon carbide neutron detectors for extreme nuclear environments, covering SiC material properties, device architectures, fabrication technologies, and radiation damage mechanisms; however, the discussion of neutron conversion layers remained largely confined to material selection rather than systematic thin-film engineering considerations [10].

For conversion-layer fabrication, Höglund et al. investigated the density, morphology, and neutron absorption properties of magnetron-sputtered B_4_C thin films in 2012, but that study focused exclusively on a single deposition route and did not provide a cross-process comparison of conversion-layer preparation strategies [11]. Massara et al. conducted a comparative study of vacuum thermal evaporation and chemical deposition for ^6^LiF conversion layers, representing one of the few attempts to evaluate different preparation strategies. Nevertheless, this comparison remained limited to the ^6^LiF system and could not be extended to other conversion materials [12]. The Microstructured Semiconductor Neutron Detector (MSND) research series led by the McGregor group provided detailed investigations into conversion-layer coating within perforated microstructures; however, the process analysis was mainly limited to mechanical backfilling of the etched trenches with enriched ^6^LiF microparticulate powder, with conformal deposition technologies such as CVD and ALD receiving insufficient attention [13].

A common structural limitation of existing reviews is that the conversion layer is typically regarded as an auxiliary detector component and discussed primarily in terms of material selection or thickness optimization, and no review has evaluated conversion layers as engineered thin-film systems through a unified framework integrating deposition processes, thin-film quality, and performance-related properties across different materials and fabrication routes. Consequently, research efforts across different fabrication routes have developed relatively independently: sputtering studies primarily investigate the influence of substrate roughness on microstructures, chemical vapor deposition (CVD) studies focus on the optimization of conformal coating parameters, and solution-based approaches emphasize binder concentration control; a unified analytical framework enabling comparison and interaction among different processing routes is still lacking.

### 1.2. Review Framework and Organization

This review is organized around a fundamental concept: the quality of a neutron conversion layer cannot be defined solely by the thermal neutron cross section of the conversion material and its geometric thickness, but should instead be characterized using four interrelated physical dimensions.

1. Areal density: the number of neutron-sensitive isotope atoms (e.g., ^10^B and ^6^Li) per unit area. It directly determines the neutron capture probability and is jointly affected by film thickness, density, and isotopic enrichment.
2. Density/uniformity: the theoretical density and microstructural uniformity of the conversion layer, which influence both the effective isotope loading and product-escape characteristics of the layer and the effective atomic areal density.
3. Adhesion/interface quality: the bonding condition between the conversion layer and the semiconductor substrate, which influences the energy loss of reaction products during passage across the interface (dead-layer effect) and the long-term reliability of the detector.
4. Conformality/step coverage (SC): the ratio of film thickness deposited on the bottom and sidewalls of a high-aspect-ratio microstructure to that on the top surface (SC = t_bottom_/t_top_). It determines whether a deposition process can coat 3D microstructured detectors uniformly and is the primary link between a deposition technology and a non-planar detector geometry.

Among the four physical dimensions, areal density is primarily determined by nuclear physics constraints and geometric factors, whereas density/uniformity, conformality/step coverage, and adhesion/interface quality are predominantly governed by the physicochemical mechanisms of the deposition process. The central premise of this review is that the performance of each deposition process across these four dimensions can be consistently understood in terms of its underlying physicochemical deposition mechanisms. For example, the line-of-sight effect inherent to physical vapor deposition (PVD) processes (e.g., magnetron sputtering and vacuum evaporation) enables high-efficiency deposition on large-area planar substrates, but also represents the physical origin of structural mismatch and limited interface quality in 3D microstructures. The surface reaction kinetics of CVD processes enable conformal coating; however, precursor choice is limited and deposition rates are relatively low, which lengthens the processing time per unit thickness; whether this bounds the attainable areal density has not been established, because areal density is not reported for CVD converters. Solution-based deposition offers good industrial scalability because it can be carried out under ambient-pressure and room-temperature conditions. However, binder dilution and interparticle porosity remain the primary factors limiting its performance.

This review does not seek to identify a universally “best” process, because the answer depends on the specific detector architecture, application scenario, and performance priorities. Instead, this review provides a process–property relationship map based on physical mechanisms and literature evidence. This map is intended to help researchers make informed process decisions according to their specific needs and identify the evidence gaps that most need to be filled.

**Figure 1:**
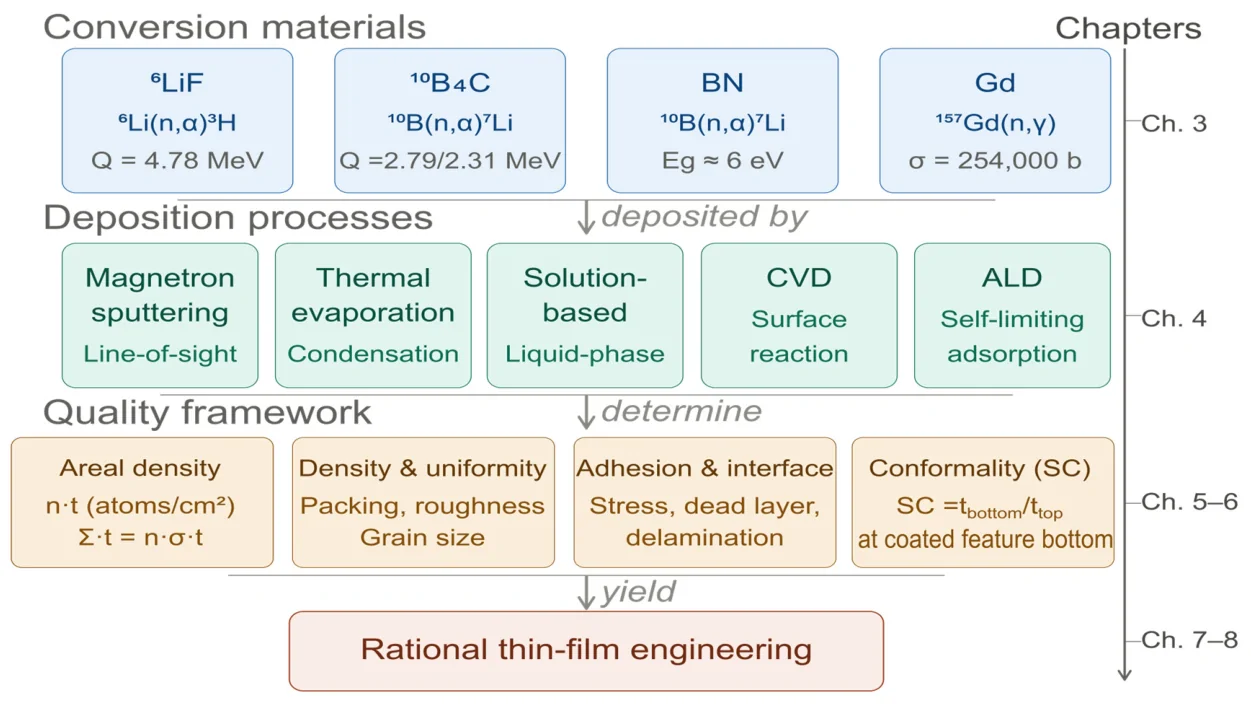
Overall logical framework of the review: neutron conversion materials (Section 3) → deposition processes (Section 4) → microstructural optimization and interface engineering (Section 5 and Section 6) → computational simulation and future perspectives (Section 7 and Section 8).

**Figure 2:**
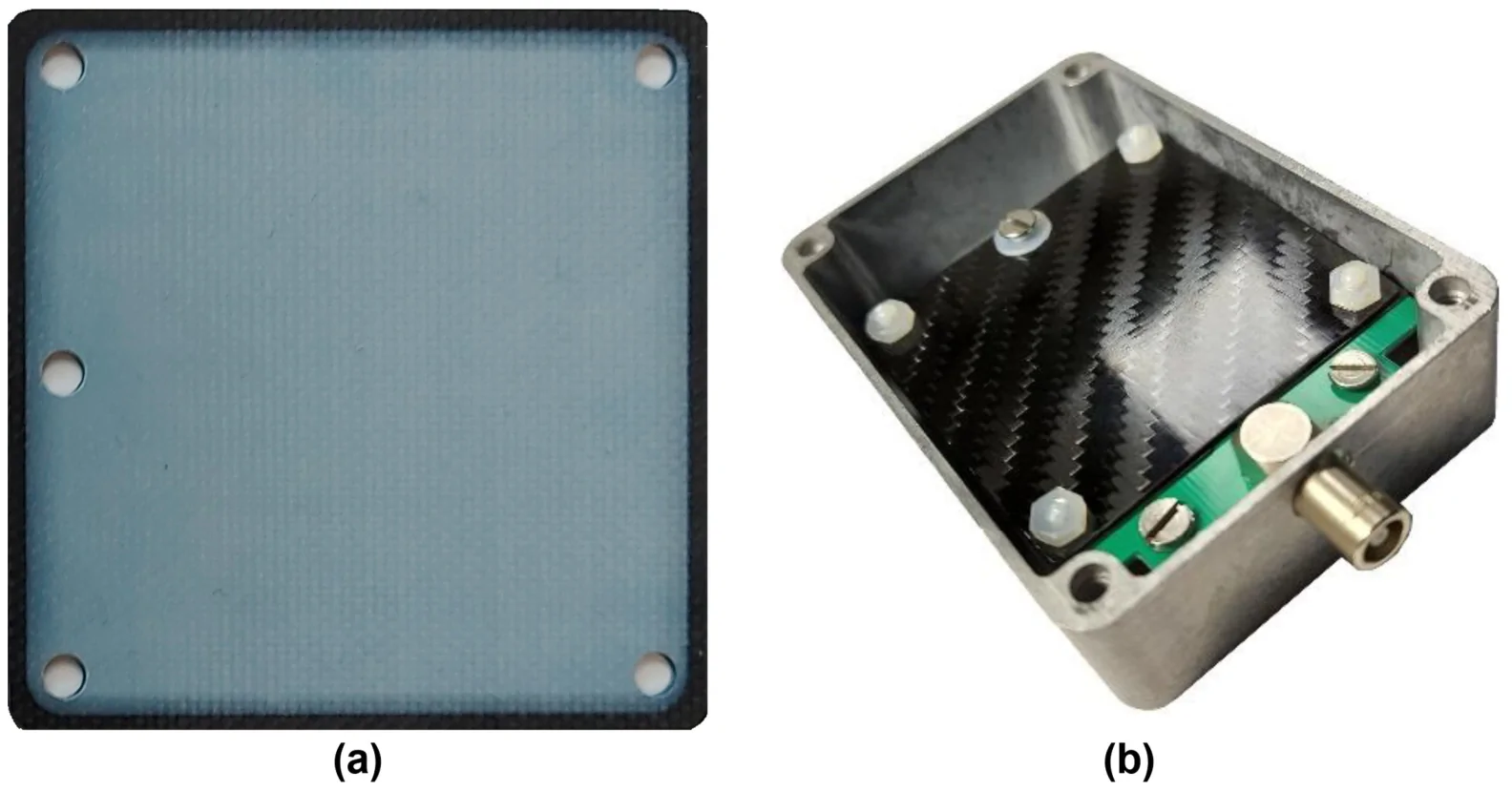
Photographs of a ^6^LiF neutron converter and of a detector based on it: (**a**) a 16 μm ^6^LiF layer deposited on a 1 mm thick carbon-fiber substrate; (**b**) an assembled SiLiF detector, in which a double-sided silicon diode is sandwiched between two such ^6^LiF-on-carbon-fiber converters inside an aluminum housing. (**a**) Reprinted from ref. [12]; (**b**) reprinted from ref. [14]. Both sources are published under the CC BY 4.0 license.

The remainder of this review is organized as follows. Section 2 derives the quality requirements for neutron conversion layers from the physical constraints imposed by nuclear reactions. Section 3 compares candidate neutron conversion materials in terms of their nuclear physics properties and process compatibility. Section 4, the core section of the review, examines the physicochemical mechanisms of five deposition processes and their performance within the four-dimensional quality framework. Section 5 discusses strategies for microstructural optimization and the compatibility between deposition processes and structural configurations. Section 6 focuses on interface engineering, a critical aspect that has long been overlooked. Section 7 reviews how computational simulation methods have been applied to neutron conversion-layer design. Section 8 examines the current data gaps in the field and discusses three key directions for future research. Section 9 presents the main conclusions of the review. Figure 1 presents the overall logical framework of the review. Figure 2 shows a finished ^6^LiF conversion layer on a carbon-fiber substrate and an assembled SiLiF detector in which two such converters sandwich a double-sided silicon diode [12,14]. Note that this review focuses on the external conversion layer of semiconductor direct-conversion detectors: scintillators (e.g., GYAGG:^6^LiF) and gas detectors (e.g., macrostructured ^10^B_4_C cathodes) are cited only in a limited way where directly relevant to process evaluation, whereas materials that are themselves neutron-absorbing semiconductors (h-BN, LiInSe_2_, Ga_2_O_3_:B, a-BC:H) operate by intrinsic conversion and therefore lie outside the scope of the external thin-film conversion layers discussed here.

## 2. Nuclear Reaction Fundamentals and Design Constraints of Neutron Conversion Layers

### 2.1. Neutron Interactions with Matter

Neutron interactions with atomic nuclei can be primarily classified into three categories: elastic scattering, inelastic scattering, and nuclear reactions involving neutron capture [3]. For semiconductor neutron detection, elastic scattering can transfer a fraction of neutron kinetic energy to recoil nuclei; however, the recoil energy is generally insufficient for effective use in semiconductor detectors, particularly for thermal neutrons where the transferred energy is nearly negligible. Inelastic scattering is also constrained by two factors: it requires incident neutron energies on the MeV scale to excite target nuclei to their first excited states, and is therefore largely unavailable in the thermal-to-epithermal neutron energy range; moreover, the subsequent de-excitation process produces gamma photons rather than charged particles, whereas the thin sensitive regions of semiconductor detectors exhibit limited photon detection efficiency and cannot readily generate distinguishable electrical signals. Consequently, nuclear reactions represent the primary mechanism capable of producing detectable charged particles or photon signals for semiconductor neutron detection. Therefore, the fundamental objective of neutron conversion-layer design in semiconductor neutron detectors is to maximize the probability that effective nuclear reaction products reach the semiconductor sensitive region under a given neutron energy spectrum.

### 2.2. Key Nuclear Reaction Parameters

Neutron conversion layers rely on isotopes with large thermal neutron capture cross sections—principally ^10^B, ^6^Li and ^157^Gd [15]—and the key nuclear reaction parameters relevant to conversion-layer design are collected in Table 1 [7,8,16,17,18]. The most widely used ^10^B(n,α)^7^Li reaction proceeds through two branches:

(1) B10+n→Li7(1.015 MeV)+α(1.777 MeV) (6%)

(2) B10+n→Li7∗(0.840 MeV)+α(1.470 MeV)+γ(0.478 MeV) (94%)

The ^6^Li(n,α)^3^H reaction has a higher Q value (4.78 MeV) [8] and yields a 2.73 MeV triton whose range in LiF is approximately 32 μm [7], which permits considerably thicker conversion layers:

(3) Li6+n→H3(2.73 MeV)+α(2.05 MeV)

### 2.3. Energy Dependence of Neutron Reaction Cross Sections

**Figure 3:**
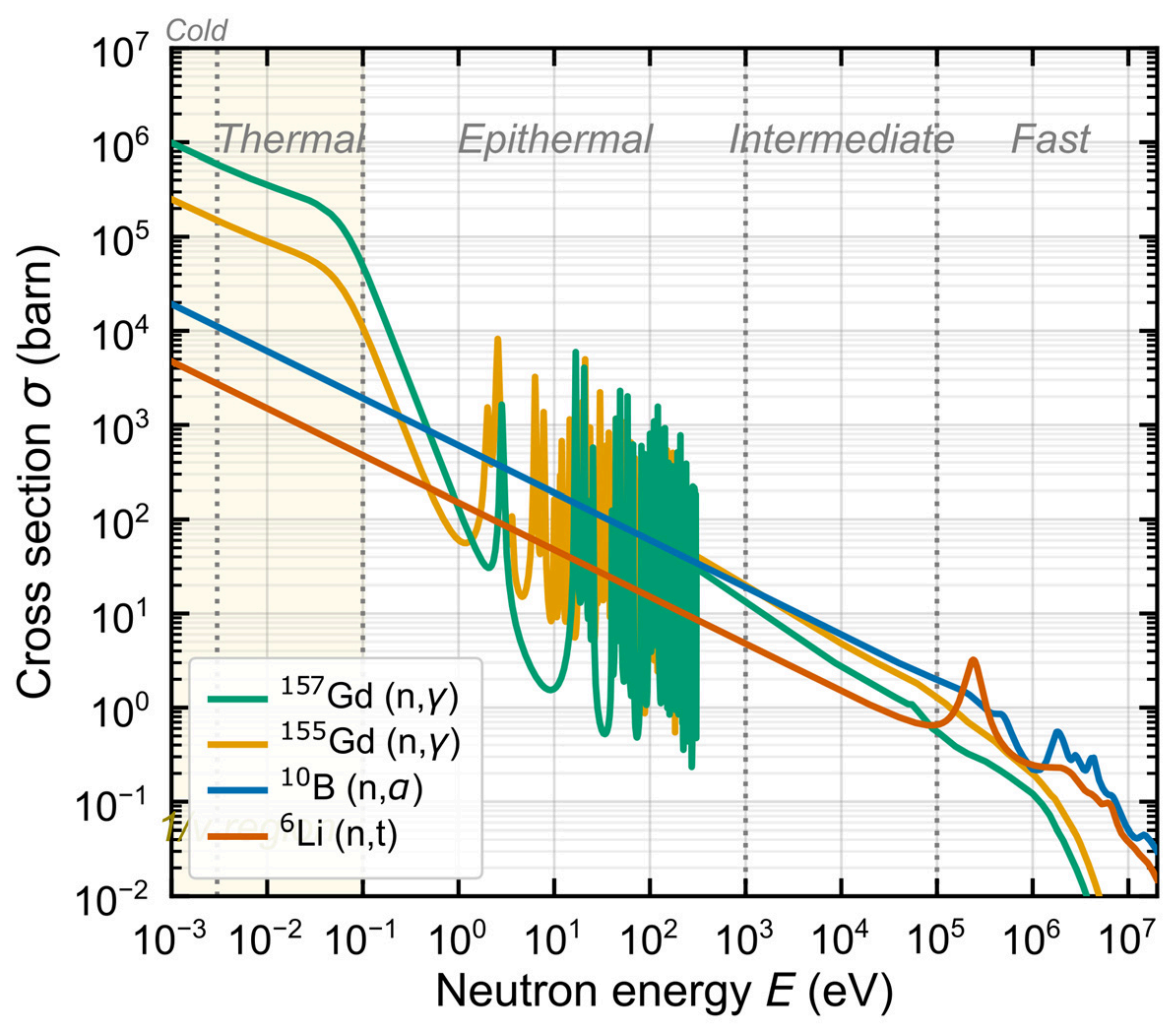
Neutron capture cross sections of four conversion isotopes (^6^Li, ^10^B, ^155^Gd, ^157^Gd) as a function of incident neutron energy (data source: ENDF/B-VIII.0 evaluated nuclear database).

In the thermal energy region (E ≈ 0.025 eV), the conversion cross sections follow the 1/v law, whose range covers most reactor neutron spectra. Gd has by far the largest thermal cross section, and although it falls off rapidly with increasing energy, its strong resolved resonances sustain appreciable values well beyond the 1/v region (Figure 3); Gd-based conversion layers are therefore also sensitive to epithermal neutrons, unlike their ^10^B- and ^6^Li-based counterparts [19,20]. Above ≈1 MeV the cross sections of all conversion isotopes drop below the barn level, so fast neutrons are detected through recoil-nucleus mechanisms or after moderation in front of the converter [21]. The energy-dependent cross sections of the four isotopes are shown in Figure 3.

### 2.4. Self-Absorption Effects and Optimal Conversion-Layer Thickness

**Figure 4:**
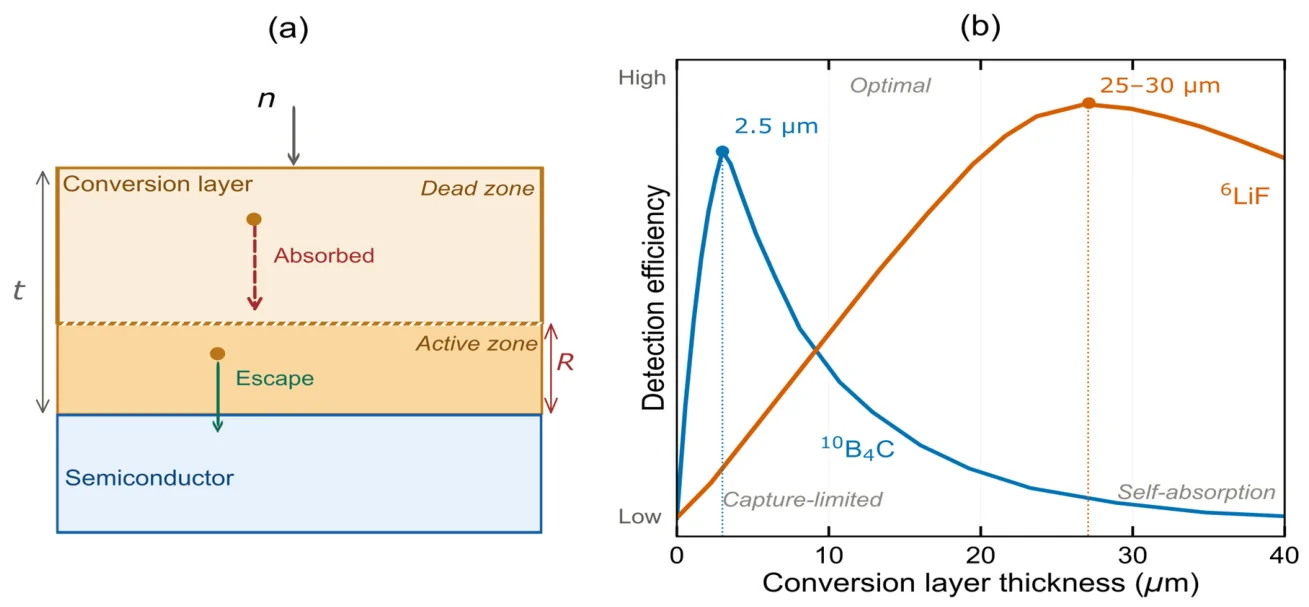
Schematic illustration of self-absorption effects and optimal conversion-layer thickness. (**a**) Transport mechanism of reaction products in the conversion layer: only products generated within a distance of R from the semiconductor interface can reach the semiconductor sensitive region and contribute to detection, while those generated beyond this effective range are absorbed by the conversion layer itself; (**b**) Dependence of detection efficiency on conversion-layer thickness. The efficiency of ^10^B_4_C peaks at approximately 2–3 μm because of the short reaction-product range, whereas ^6^LiF reaches its maximum at approximately 25–30 μm due to the much longer product range [17,22]. These optima refer to a single-sided, single-layer planar converter under front irradiation, and the associated intrinsic efficiency ceiling of approximately 4–5% at a 300 keV lower-level discriminator applies to that configuration rather than to planar detectors in general (Section 5.2). This schematic illustration is qualitative, and the vertical axis does not indicate absolute efficiency values.

Reaction products have finite ranges in the converter—approximately 3.5 μm for the 1.47 MeV α and 1.6 μm for the 0.84 MeV ^7^Li in ^10^B_4_C, and approximately 32 μm for the 2.73 MeV triton in LiF [7]—so only products generated within roughly one range of the semiconductor interface can escape and contribute a signal (Figure 4a). Self-absorption therefore makes the detection efficiency a non-monotonic function of conversion-layer thickness: it increases with neutron capture up to an optimum—approximately 2–3 μm for ^10^B-based layers and 25–30 μm for ^6^LiF in the single-sided, single-layer, front-irradiation planar geometry assumed throughout this section, in which neutrons enter through the conversion layer at a 300 keV lower-level discriminator (Figure 4b) [17,22]—and decreases thereafter, when the additional capture no longer compensates for the products absorbed inside the converter. Quantitative escape-probability descriptions were established analytically for front-, back-coated, and sandwich geometries [7], extended by Monte Carlo to perforated and grooved microstructures [23], and applied to stacked and trench configurations [24]; the resulting efficiency ceilings, together with their dependence on geometry, discriminator setting, and layer stacking, are discussed in Section 5.2.

### 2.5. From Nuclear Physics Constraints to Conversion-Layer Quality Requirements

The self-absorption effect primarily governs the areal density dimension, whereas the requirements for density/uniformity and adhesion/interface quality arise not from nuclear reaction physics itself, but from the physicochemical mechanisms of deposition processes and interface engineering. Therefore, these aspects are discussed in later sections rather than here.

These physical constraints naturally translate into four key quality requirements for neutron conversion-layer films. High areal density requires sufficient converter isotope content to maximize neutron capture probability, but its increase is ultimately limited by self-absorption effects. Density/uniformity governs the energy-loss characteristics of reaction products within the conversion layer. Porous or low-density films reduce the effective areal density of converter isotopes achievable at a given geometrical thickness, thereby lowering the neutron capture probability; at the same time, the reduced stopping power extends the geometrical range of reaction products and can even facilitate their escape. The net efficiency penalty of porosity is therefore dominated by the loss of isotope loading rather than by increased product energy loss. Adhesion/interface quality controls the energy loss of reaction products crossing the conversion-layer–semiconductor interface, where defective interfacial layers may introduce “dead layers”, and directly influences long-term device reliability. These four dimensions establish the fundamental conceptual framework underlying all subsequent analyses in this review.

## 3. Comparative Analysis of Neutron Conversion Materials

The selection of candidate neutron conversion materials is governed by the interplay of three major constraints. (1) Nuclear physics properties: thermal neutron cross section, reaction product characteristics, and Q value define the theoretical limit of conversion efficiency. (2) Chemical and physical stability: environmental stability, temperature tolerance, and radiation resistance determine long-term application feasibility. (3) Process compatibility: thin-film processability, thermal expansion matching with semiconductor substrates, and chemical inertness define the accessible fabrication strategies. This section evaluates the major candidate materials within this three-constraint framework.

### 3.1. ^6^LiF: The Established Reference Material for Neutron Conversion Layers

^6^LiF remains the most widely adopted neutron conversion-layer material in semiconductor neutron detectors. The primary advantages of ^6^LiF originate from the high Q value (4.78 MeV) [8] of the ^6^Li(n,α)^3^H reaction and the long range of its reaction products (approximately 32 μm for ^3^H in LiF). These features allow efficiency enhancement over a broader thickness range and help reaction products penetrate potential interfacial dead layers [7,12].

In practical devices, ^6^LiF has been successfully integrated with multiple semiconductor platforms. Hong et al. demonstrated the feasibility of a 4H-SiC detector coated with a 25 μm-thick ^6^LiF layer (95% ^6^Li enrichment) for neutron flux monitoring in the D-D fusion neutron environment of the EAST tokamak. The detector exhibited reliable and stable performance throughout the entire experimental campaign [25]. Liu et al. demonstrated neutron detection from ^241^Am-Be and ^252^Cf sources using a single-crystal diamond detector coated with a 5 μm-thick ^6^LiF conversion layer [26]. Shi et al. deposited a 25 μm-thick ^6^LiF conversion layer on SiC substrates via electron-beam evaporation and achieved a linear response across a neutron fluence rate range of 1 × 10^3^–1 × 10^6^ cm^−2^·s^−1^ [27]. Massara et al. compared vacuum thermal evaporation and chemical deposition for ^6^LiF layer fabrication [12]. Thermal evaporation produced high-purity films with good adhesion, while chemical deposition provided a simpler and lower-cost approach but generally suffered from reduced Density and Uniformity.

The characterization of film quality in the existing ^6^LiF literature remains much less developed than that for ^10^B_4_C. Most studies report only film thickness and detection efficiency, while systematic evaluations of density, roughness, adhesion, and other film-quality parameters are still scarce. The major limitations of ^6^LiF are the low natural abundance of ^6^Li (~7.5%), the resulting requirement for costly isotopically enriched ^6^LiF (typically ≥95% enrichment), and the intrinsic hygroscopicity of LiF, which necessitates encapsulation for long-term operational stability.

### 3.2. ^10^B and ^10^B_4_C: High-Performance Conversion Materials with Comprehensive Film Characterization

The thermal neutron cross section of ^10^B (3840 barn) is approximately four times higher than that of ^6^Li (940 barn). The natural abundance of ^10^B in boron is approximately 20%, resulting in lower raw material costs compared with highly enriched ^6^LiF. Among boron-containing conversion materials, ^10^B_4_C is currently the most actively investigated system and is the best-characterized conversion-layer material in terms of thin-film quality. This advantage reflects the research experience accumulated from long-term use of such films as hard coatings.

Zhu et al. developed an industrial-scale magnetron sputtering system capable of depositing large-area uniform ^10^B_4_C films with dimensions of 1500 × 400 mm^2^, achieving a thickness non-uniformity better than ±3%. A 3 μm-thick ^10^B_4_C film used as the cathode of a ceramic gas electron multiplier (GEM) gas detector exhibited an experimentally measured detection efficiency of 3.3 ± 0.3% for 25 meV neutrons, which agreed well with the Geant4 simulation result of 3.4% [28]. Clement et al. developed a Ti–B–Ni multilayer ^10^B conversion structure to meet the specific requirements of ultracold neutron detection. Using a microwave plasma-assisted co-sputtering method, they achieved high detection efficiency over a broad ultracold neutron velocity range on silicon CCD sensors [29]. Control of the B/C stoichiometric ratio in ^10^B_4_C films remains a fabrication challenge. Zhu et al. reported a B/C ratio of 5.18 in sputtered films, deviating from the ideal stoichiometric value of 4.0 [28]. At the device level, Sun et al. fabricated SiC neutron detectors based on boron carbide conversion layers in 2025 and experimentally characterized their performance, demonstrating the feasibility of the ^10^B_4_C/SiC detector system [30].

^10^B_4_C is currently the best-characterized neutron conversion material in terms of thin-film quality. Its fundamental physical limitations, including the optimal thickness and self-absorption limit, have been largely established. These limits are configuration-specific rather than material-intrinsic: as quantified in Section 5.2, they apply to a single-sided, single-layer planar converter at a 300 keV lower-level discriminator and shift with geometry, discriminator setting and layer stacking. Three-dimensional microstructures offer the largest additional margin, but they are one route among several rather than the sole means of exceeding this ceiling.

### 3.3. BN: An Emerging Wide-Bandgap-Compatible Conversion Material

Hexagonal boron nitride (h-BN) has attracted considerable attention due to its unique dual functionality. It can provide neutron conversion capability through the ^10^B(n,α)^7^Li reaction, while its wide bandgap of approximately 6 eV enables direct charge collection. Therefore, h-BN has the potential to simultaneously serve as both the neutron conversion layer and the semiconductor sensitive region, a feature rarely observed among other candidate conversion materials.

For detection performance, Maity et al. demonstrated in 2020 that h-BN devices (an intrinsic-conversion semiconductor, included here only for cross-process comparison) reached a normalized thermal neutron detection efficiency of 58.9 ± 2.4% over an area of 1 cm^2^ by improving the electron mobility–lifetime product of epitaxial h-BN films by a factor of six and reducing the surface recombination field by a factor of three. This remains the highest efficiency reported for h-BN neutron detectors, and one of the few high-efficiency records among conversion-material-based semiconductor neutron detectors [31]. The key message: material defect density, rather than nuclear physics, is the dominant factor restricting h-BN detection efficiency. With further improvements in material quality, the achievable efficiency of h-BN may substantially exceed the values currently reached.

For process feasibility, Kim et al. demonstrated in 2023 the conformal growth of few-layer h-BN films on high-aspect-ratio (~7:1) silicon nanogrooves using pulsed metal–organic chemical vapor deposition (MOCVD). X-ray absorption fine structure (XAFS) spectroscopy combined with density functional theory (DFT) calculations revealed that B–O bonds on non-catalytic SiO_2_ surfaces act as critical nucleation sites for h-BN growth [32]. This result indicates that h-BN films can, in principle, be conformally deposited on 3D microstructures through CVD-based processes, providing a process feasibility foundation for their application in grooved or perforated neutron detector architectures.

h-BN offers unique functional integration potential among candidate conversion materials, but it also faces a dual constraint. Its detection performance is governed simultaneously by nuclear physics processes (the ^10^B reaction cross section and reaction-product self-absorption) and by extreme sensitivity to material defects. The latter is currently the dominant bottleneck determining the achievable efficiency limit. Therefore, controlled epitaxial growth of high-quality h-BN films and their process compatibility with microstructured detector architectures remain the key challenges toward practical implementation of this technology.

### 3.4. Gd-Based Materials: High-Cross-Section Neutron Converters

The most prominent feature of Gd-based conversion layers is their extremely high thermal neutron absorption cross sections. The absorption cross sections of ^155^Gd and ^157^Gd are 60,900 barn and 254,000 barn, respectively, representing the highest values among all stable nuclides. This cross-section advantage allows Gd-based conversion layers to capture neutrons efficiently at sub-micrometer thicknesses, an asset where detector thickness or weight is constrained, but the nuclear reaction physics of Gd also introduces challenges associated with its high absorption cross section, limiting its practical application range.

Dumazert reviewed the applications of Gd in neutron detection, with a detailed discussion of the cascade radiation processes following neutron capture, including the energy distributions and branching ratios of internal conversion electrons, Auger electrons, and characteristic γ-rays. The requirements of different detector architectures for Gd conversion layers, as well as key performance parameters such as neutron sensitivity, γ-ray rejection ratio, and mixed-field detection limits, were summarized [18]. This review remains the fundamental reference for understanding the physical mechanisms of Gd-based conversion layers.

For thickness optimization, Bodie and Barnett developed a dedicated Monte Carlo model in 2024 to simulate neutron absorption in natural Gd and the subsequent photon and electron emission processes, quantitatively determining the optimal Gd thickness for thermal neutron detection [19]. In 2025, Kröhnke further presented an optimal thickness analysis for isotropic thermal neutron fields in his doctoral dissertation, providing quantitative guidance for the engineering design of Gd conversion layers [33].

For device applications, Gimenez et al. embedded Gd_2_O_3_ particles into photoresist as a neutron conversion layer and coated it onto a field oxide field-effect transistor (FOXFET) for n-γ mixed-field dose measurements in boron neutron capture therapy (BNCT) in 2022 [20]. This work demonstrated the feasibility of Gd-based conversion layers for mixed radiation field dosimetry applications.

Gd-based materials have the largest thermal neutron cross sections, but their nuclear reaction characteristics impose limitations in applications requiring precise γ-ray discrimination, and device-level experimental evidence for semiconductor neutron detectors based on Gd conversion layers remains limited. Therefore, Gd-based conversion layers are more suitable for specific application scenarios where γ-ray discrimination requirements are moderate and strict constraints exist on detector thickness, such as mixed-field dosimetry and ultrathin conversion screens for imaging systems. They are less competitive than ^10^B_4_C or ^6^LiF as general-purpose thermal neutron conversion materials.

### 3.5. Material-Process Compatibility of Neutron Conversion Layers

Based on the physicochemical properties of the above materials and existing literature, intrinsic compatibility relationships exist between different conversion materials and deposition processes:

1. ^6^LiF: Vacuum evaporation (in practice the thermal variant) is the most suitable fabrication approach, which is determined by its vapor pressure characteristics and chemical compatibility with the evaporation environment. ^6^LiF can also be prepared through chemical deposition methods, but it is generally less suitable for reactive sputtering processes.
2. ^10^B_4_C: Magnetron sputtering is the most appropriate deposition technique because it enables large-area uniform deposition and has already achieved industrial-level validation. Electron-beam evaporation is also applicable, while solution-based methods such as spin coating can be employed for fabricating microstructured neutron detectors.
3. BN: MOCVD and plasma-enhanced chemical vapor deposition (PECVD) represent key approaches for achieving high-quality BN films. Its capability of conformal growth provides a unique advantage for integration with 3D microstructured detectors.
4. Gd/Gd_2_O_3_: These materials can be fabricated through electron-beam evaporation or solution-based coating methods, such as embedding Gd-containing particles into polymer matrices.

The material-process compatibility relationships above mainly reflect the current level of research accumulation reported in the literature rather than fundamental physical limitations. With the continued development of thin-film technologies, new combinations of conversion materials and fabrication processes may prove feasible in the future.

## 4. Deposition Technologies and Their Impact on Conversion-Layer Film Quality

This section is the central focus of this review. The physicochemical mechanisms of five representative deposition technologies, including magnetron sputtering, vacuum evaporation, chemical vapor deposition (CVD), atomic layer deposition (ALD), and solution-based methods, are analyzed. Particular emphasis is placed on how the inherent characteristics of each deposition process determine the resulting conversion-layer quality, leading to predictable advantages and trade-offs among four critical dimensions (areal density, density/uniformity, conformality, and adhesion/interface quality).

### 4.1. Magnetron Sputtering

#### 4.1.1. Physical Mechanism

Magnetron sputtering uses Ar^+^ ions generated by glow discharge to bombard the target surface under the influence of orthogonal electric and magnetic fields, inducing the ejection of target atoms and their subsequent deposition onto the substrate. The fundamental physical characteristics of magnetron sputtering include:

1. Moderate kinetic energy of sputtered atoms: The kinetic energy of sputtered atoms is typically in the range of approximately 1–10 eV, which is higher than that of thermally evaporated atoms (~0.1 eV) but considerably lower than that achieved in pulsed laser deposition. Therefore, sputtered atoms retain a certain degree of surface migration capability after arriving at the substrate, promoting atomic rearrangement and film densification.
2. Line-of-sight effect: The substrate must have direct visibility of the target to achieve uniform deposition. This geometric limitation is the fundamental reason why magnetron sputtering exhibits excellent thickness uniformity on planar substrates but relatively poor conformality on 3D structures.
3. Magnetic-field-enhanced plasma generation: The introduction of a magnetic field confines electron trajectories near the target surface, increasing electron-gas collision probability and thereby enhancing plasma density and deposition rate.

**Figure 5:**
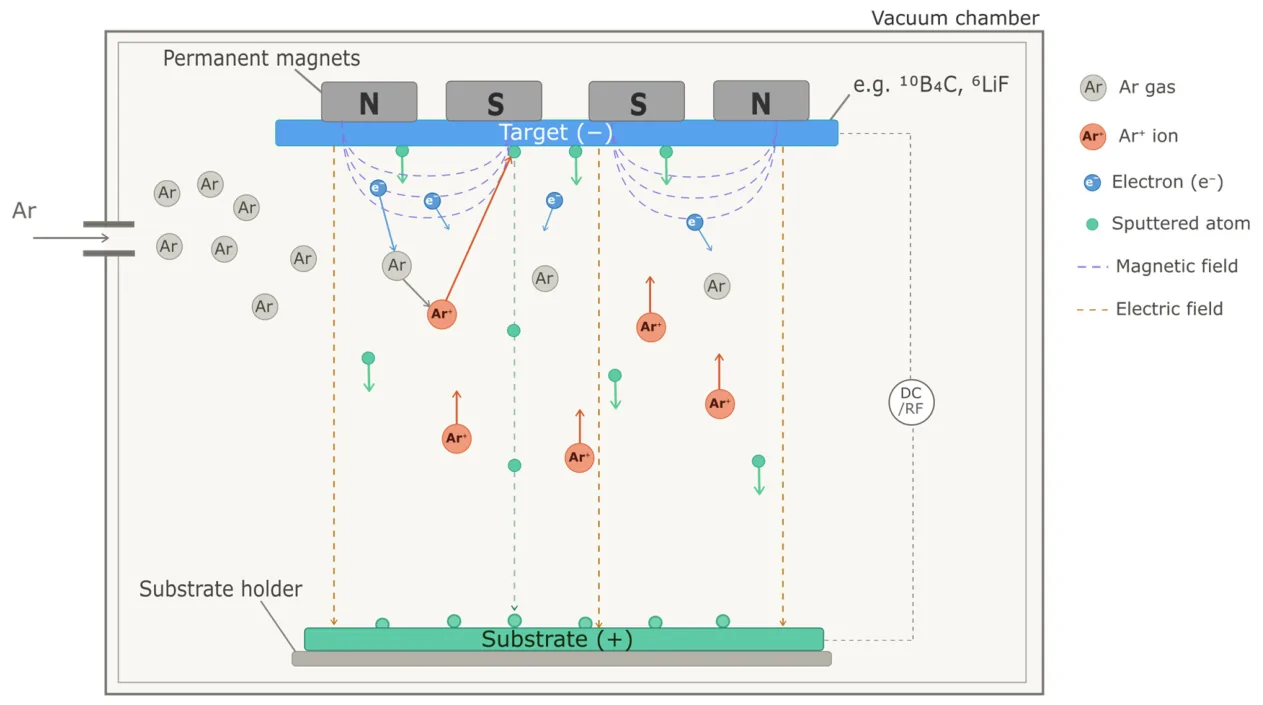
Schematic diagram of the magnetron sputtering process. Ar gas is ionized into Ar^+^ ions in an orthogonal electromagnetic field and accelerated to bombard the target (e.g., ^10^B_4_C or ^6^LiF), leading to sputtering and deposition of target atoms onto the substrate. The magnetic field confines electron trajectories to enhance plasma density and deposition efficiency.

A schematic illustration of the magnetron sputtering process is shown in Figure 5.

Magnetron sputtering is currently the most extensively studied and well-documented deposition technology for the fabrication of neutron conversion layers. Zhu et al. developed an industrial-scale magnetron sputtering system capable of depositing large-area uniform ^10^B_4_C films with dimensions of 1500 × 400 mm^2^ [28]. Clement et al. addressed the specific requirements of ultracold neutron detection by fabricating a Ti-B-Ni multilayer ^10^B conversion structure using microwave plasma-assisted co-sputtering. The resulting detector achieved high detection efficiency over a broad range of ultracold neutron velocities [29].

In addition, various sputtering configurations have been explored for neutron conversion-layer fabrication. In 2012, Höglund et al. investigated the density, morphology, and neutron absorption characteristics of ^10^B_4_C films prepared by direct-current magnetron sputtering (DCMS) [11]. In 2016, Schmidt et al. compared the effects of DCMS and high-power impulse magnetron sputtering (HiPIMS) on the properties of ^10^B_4_C thin films [34]. In 2021, Shin et al. investigated the influence of inclined-substrate sputtering geometry on the microstructure and residual stress of ^10^B_4_C films [35].

#### 4.1.2. Impact on the Four Quality Dimensions of Conversion Layers

1. Areal Density (Excellent, Demonstrated): The high deposition rate of magnetron sputtering enables efficient fabrication of micrometer-scale conversion films. Zhu et al. reported that a 3 μm-thick ^10^B_4_C film deposited by magnetron sputtering and used as the cathode of a ceramic gas electron multiplier (GEM) gas detector achieved an experimentally measured thermal neutron detection efficiency of 3.3 ± 0.3% for 25 meV neutrons, which was in excellent agreement with the Geant4 simulation result of 3.4%. The study further characterized the film composition, reporting a B/C atomic ratio of 5.18, an oxygen content of 6 at.%, and a nitrogen content of 0.79 at.% [28]. Multiple independent experimental and simulation studies have consistently confirmed that the optimal thickness of ^10^B_4_C conversion layers is approximately 2–3 μm, and magnetron sputtering can provide reliable control over the effective areal density within this thickness range. At the 3 μm anchor of [28], this corresponds to a ^10^B areal density of ≈5.6 × 10^2^ μg/cm^2^, ≈100% of the value at the 2.5 μm optimum for elemental ^10^B (Table 2).
2. Density and Uniformity (Good, Demonstrated): The density of sputtered films is strongly dependent on process parameters, including sputtering pressure, target-to-substrate distance, and substrate bias voltage. Schmidt et al. reported densities of 2.2–2.3 g/cm^3^ for ^10^B_4_C films deposited by both DCMS and HiPIMS [34]. According to the Thornton-modified Movchan-Demchishin structure zone model (SZM), magnetron sputtering generally occurs in the low-temperature regime with a normalized temperature ratio of Ts/Tm < 0.3, where atomic surface diffusion is limited. As a result, the deposited films tend to exhibit a columnar grain structure, in which grain boundaries become potential weak regions affecting film densification. In 2021, Shin et al. demonstrated that substrate inclination could effectively regulate film microstructure and achieve nearly zero residual stress at specific inclination angles [35]. In 2022, Bayu Aji et al. investigated the influence of substrate temperature on the residual stress of ^10^B_4_C films and identified 185–250 °C as the optimal temperature range for growing films with near-zero internal stress [36]. For large-area uniformity, Zhu et al. achieved thickness uniformity better than ±3% over an area of 1500 × 400 mm^2^ in 2023, which is among the best large-area uniformity reported for conversion-layer films [28].
3. Adhesion and Interface Quality (Good, Demonstrated): Lai et al. measured the adhesion of DCMS-deposited ^10^B_4_C films on Si, Al, Ti, and Cu substrates using a cross-cut tape test with a self-defined 0–5 residual-area scale (0 = coating fully removed, 5 = coating fully retained); no standard was cited in [37]. Almost all samples graded 5, and the few lower grades occurred only for stacks containing a Ti or Cu buffer layer, where the coating was removed at the metal/Si interface rather than at the B_4_C interface. This remains one of the few dedicated adhesion studies of neutron conversion films [37]. Zhang C. et al. compared two detector configurations: a direct-contact deposition configuration, in which ^10^B_4_C was directly sputtered onto the surface of silicon detectors, and an indirectly coupled configuration, in which ^10^B_4_C was first deposited on an aluminum substrate and subsequently bonded to the silicon detector. The indirectly coupled configuration exhibited superior performance, suggesting that direct sputtering of conversion layers onto semiconductor surfaces may introduce unfavorable interfacial effects. However, the underlying mechanisms, including sputtering-induced damage, interfacial oxidation, and thermal stress, have not yet been independently clarified [38].
4. Conformality (Poor, Demonstrated): Magnetron sputtering exhibits line-of-sight transport, which fundamentally limits its capability to coat 3D microstructures uniformly. Quantitative step-coverage data for sputtered ^10^B_4_C films remain scarce, one example being the study of Schmidt et al., who deposited films by DCMS and HiPIMS in 2.1 mm-deep grooves with a 45° opening (Ar at 500–800 mPa) and obtained a bottom step coverage of 0.23–0.27 and a slope step coverage of 0.60–0.70 [34]; the bottom value lies below the SC ≥ 0.5 lower bound of the Moderate tier, placing magnetron sputtering in the Poor tier defined in the note to Table 2. Elevated-pressure operation, HiPIMS, and dual-axis substrate rotation can marginally improve sidewall deposition but do not change the underlying line-of-sight limitation.

#### 4.1.3. Advantages and Limitations

Magnetron sputtering is the most mature industrial-scale technology for neutron conversion-layer fabrication, with its key strength being the capability of producing highly uniform films over large areas. However, its line-of-sight deposition mechanism limits conformal coating on 3D microstructures. In addition, the direct relationship between interfacial adhesion properties and detector-level performance remains insufficiently understood, an evidence gap that must be closed before sputtered conversion layers reach practical semiconductor neutron detector applications.

### 4.2. Vacuum Evaporation (Thermal/Electron-Beam)

#### 4.2.1. Physical Mechanism

In vacuum evaporation, the source material is heated to its evaporation temperature, generating vapor-phase atoms or molecules that subsequently condense on the substrate surface with extremely low kinetic energies (~0.1 eV). The heating method defines two variants: thermal evaporation, in which a resistive boat or crucible heats the source, and electron-beam evaporation, in which a focused electron beam does so. Both share the same vapor-transport and film-growth physics and differ mainly in the source temperature that can be reached, which is why refractory source materials require the electron-beam variant, and in cost. In this review, “vacuum evaporation” is used as the umbrella term, and the specific variant is named wherever the distinction matters or wherever the cited study used only one of them. Due to the low kinetic energy of evaporated species, film growth is primarily governed by thermal diffusion on the substrate surface rather than subsurface implantation or energetic atomic bombardment.

In addition, the evaporation process exhibits an inherent selective separation effect for impurities, which is beneficial for achieving high-purity thin films. Compared with energetic deposition techniques such as magnetron sputtering, vacuum evaporation provides a low-damage deposition environment, making it particularly suitable for conversion materials requiring high chemical purity and minimal substrate modification.

**Figure 6:**
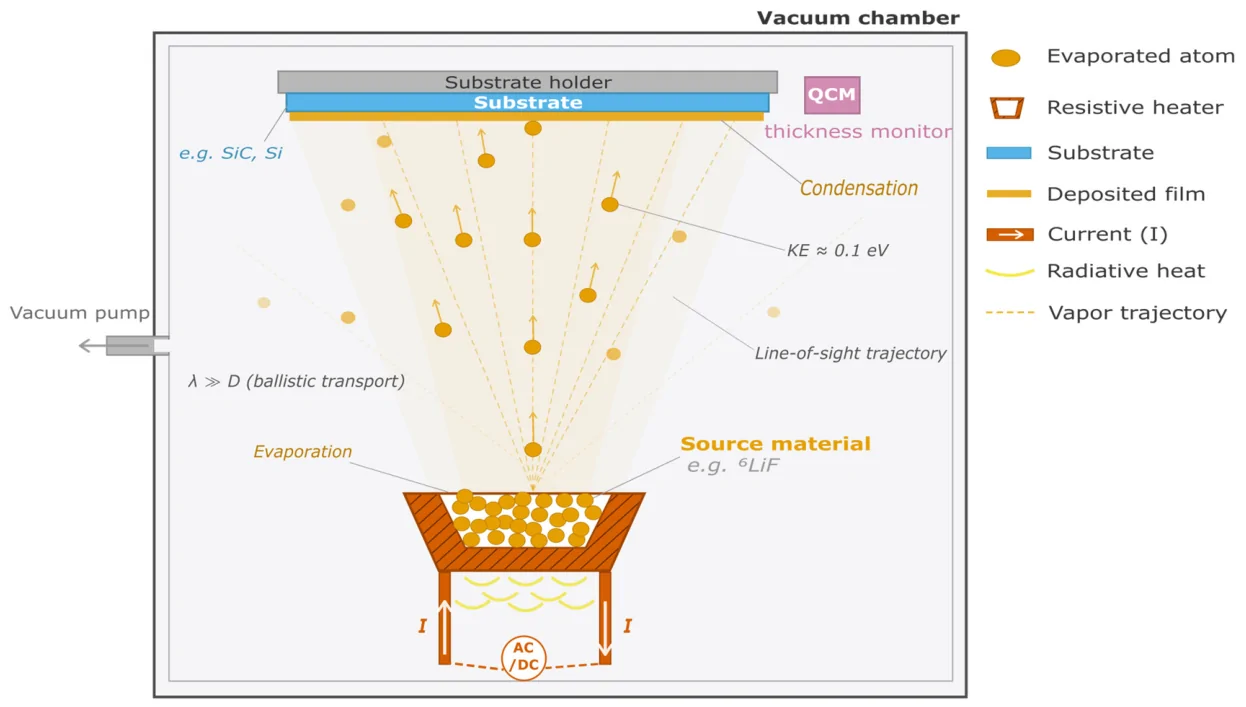
Schematic illustration of the thermal evaporation process. Inside the vacuum chamber, the low-melting-point source material (e.g., ^6^LiF) is heated by a resistive heating boat to the evaporation temperature (refractory materials such as ^10^B_4_C instead require electron-beam evaporation). The evaporated atoms undergo ballistic transport along the line-of-sight direction and condense on the substrate surface with low kinetic energies (~0.1 eV) to form a thin film. A vacuum pump maintains the low-pressure deposition environment, while a quartz crystal microbalance (QCM) enables real-time monitoring of deposition rate and film thickness.

A schematic illustration of the thermal variant is shown in Figure 6.

Vacuum evaporation has been widely applied for the fabrication of neutron conversion layers, particularly for ^6^LiF-based systems. Massara et al. compared vacuum thermal evaporation and chemical deposition methods for preparing ^6^LiF conversion layers, describing the fabrication procedures, advantages, and limitations of both approaches [12]. Almaviva et al. deposited an Al contact electrode followed by a ^6^LiF conversion layer onto CVD diamond detectors using thermal evaporation, demonstrating the feasibility of integrating evaporated conversion films with semiconductor neutron detectors [21].

In 2019, Liu et al. coated single-crystal diamond detectors with a 5 μm-thick ^6^LiF layer enriched to 95% in ^6^Li [26]. In 2022, Hong et al. employed 4H-SiC detectors coated with a 25 μm-thick ^6^LiF layer (95% ^6^Li enrichment) for D-D fusion neutron diagnostics in the EAST tokamak device [25]. In 2024, Shi et al. deposited 25 μm-thick ^6^LiF films with 95% ^6^Li enrichment onto SiC substrates using electron-beam evaporation-based vacuum deposition technology [27]. Zhang C. et al. also used electron-beam evaporation to deposit ^10^B_4_C films on different substrates, including aluminum and glass, in 2023 [38].

#### 4.2.2. Impact on the Four Quality Dimensions of Conversion Layers

1. Areal Density (Excellent, Demonstrated): Vacuum evaporation (thermal or electron-beam) can effectively deposit ^6^LiF films with thicknesses of up to several tens of micrometers, matching the approximately 32 μm long range of ^3^H reaction products from the ^6^Li(n,α)^3^H reaction in LiF. Shi et al. deposited 25 μm-thick ^6^LiF (95% ^6^Li) on SiC substrates by electron-beam evaporation and achieved a good linear response within a neutron flux range of 1 × 10^3^–1 × 10^6^ cm^−2^·s^−1^ [27]; taking this as the representative anchor gives a ^6^Li areal density of ≈1.45 × 10^3^ μg/cm^2^, matching the ≈25 μm optimum. Liu et al. detected neutron signals from ^241^Am–Be and ^252^Cf sources using a 5 μm-thick ^6^LiF/single-crystal diamond detector [26], and Hong et al. reported a 25 μm-thick ^6^LiF/4H-SiC detector that remained stable throughout the EAST experimental campaign [25] (the deposition route is not specified in that work); these correspond to ≈20% and ≈100% of the reference value, respectively (Table 2).
2. Density and Uniformity (Moderate, Expected): The extremely low kinetic energy of evaporated atoms (~0.1 eV) causes films to tend to grow in a columnar crystal mode, and the gaps between columnar crystals reduce the film density. This is a general physical characteristic of thermally evaporated films, but in the neutron conversion layer ^6^LiF system, the literature directly reporting density data is extremely scarce, in sharp contrast to the abundant density characterization available for sputtered ^10^B_4_C systems. The large-area uniformity is limited by the geometric arrangement of the evaporation source, and the line-of-sight path of the evaporation source determines the thickness distribution. Massara et al. pointed out that ^6^LiF films prepared by chemical deposition methods generally exhibit inferior Density and Uniformity compared with those prepared by vacuum evaporation [12].
3. Adhesion/Interfacial Quality (Good, Expected): The bonding between low-energy evaporated atoms and the substrate mainly relies on van der Waals forces rather than chemical bonding, resulting in generally weaker adhesion compared with sputtering and CVD. Massara et al. reported that thermally evaporated ^6^LiF films showed quite good adhesion on multiple substrates including Si. No quantitative adhesion test (cross-cut or pull-off) was performed on these layers [12]. The lattice mismatch and differences in thermal expansion coefficients between ^6^LiF and semiconductor substrates such as SiC, Si, and diamond further aggravate this issue. In contrast to sputtered ^10^B_4_C systems, where dedicated cross-cut adhesion measurements have already been reported (Lai et al., 2022 [37]) quantitative evidence for the adhesion performance of evaporated ^6^LiF films remains particularly limited.
4. Conformality (Poor, Expected): Vacuum evaporation transports vapor atoms ballistically along the line-of-sight direction, so sidewall and bottom coverage on 3D microstructures is expected to be near zero in the absence of substrate rotation. No quantitative step-coverage ratio of an evaporated ^6^LiF or ^10^B_4_C conversion layer has been reported in the neutron-detection literature; planetary or dual-axis substrate rotation can improve sidewall deposition but is incompatible with the simple resistively heated boat geometry used in most ^6^LiF thermal-evaporation studies to date, so conformal coating on 3D microstructures is not a realistic capability of vacuum evaporation at the present state of the art.

#### 4.2.3. Advantages and Limitations

Within this family, thermal evaporation is the preferred fabrication method for ^6^LiF conversion layers. It can deposit thick films (≥25 μm) on various semiconductor platforms (SiC, diamond, and Si), matching the long range of ^3^H. It has been validated in multiple realistic neutron environments, including the EAST tokamak and Am-Be/Cf-252 source tests. The main limitations originate from the intrinsic columnar microstructure of low-energy deposition and the near absence of thin-film quality characterization (density and adhesion) in the literature. The latter is the largest gap between thermally evaporated ^6^LiF systems and sputtered ^10^B_4_C systems.

### 4.3. Solution-Based Methods

#### 4.3.1. Physical Mechanism

**Figure 7:**
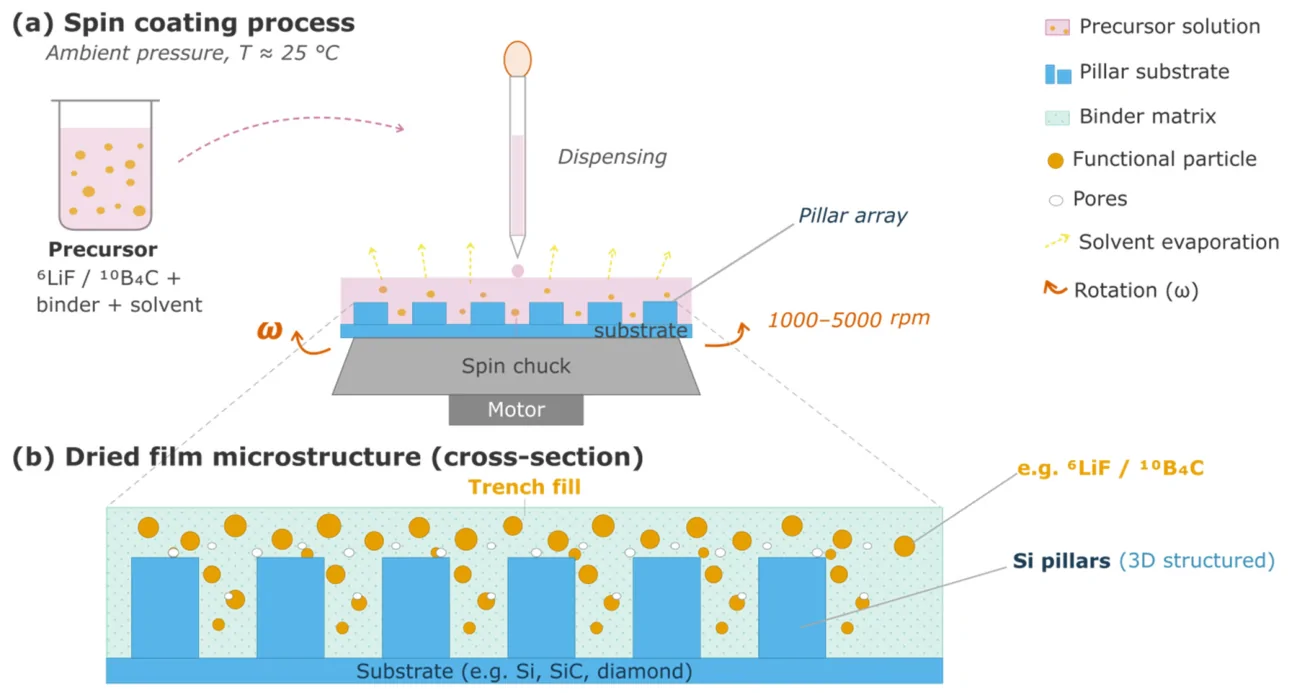
Schematic illustration of the solution-based process. (**a**) Spin-coating process: A precursor solution containing ^6^LiF/^10^B_4_C particles and binder is dispensed onto a rotating pillar-array substrate (1000–5000 rpm). The liquid phase spreads under centrifugal force and fills the 3D microstructures, while the solvent evaporates simultaneously. (**b**) Microscopic cross-section of the dried film: Functional particles, binder matrix, and pores form a composite structure, uniformly covering the tops of pillars and the grooves.

Solution-based methods use liquid-phase precursors (suspensions containing target nuclide particles or precursor solutions) to form films on substrates through techniques such as spin coating, blade coating, or inkjet printing, followed by drying or curing treatment. The key characteristics of these methods include operation under ambient pressure and temperature conditions and the fluidity of the liquid phase, which enables the filling of grooves or perforated structures. After drying, the resulting film consists of functional particles dispersed within a binder (usually polymer) matrix. A schematic illustration of the solution-based process is shown in Figure 7.

In 2022, Fedorov et al. mixed GYAGG scintillator powder and ^6^LiF particles with binder and deposited the mixture onto an aluminum substrate to fabricate a composite scintillation screen for neutron detection [39]; although this particular device is a scintillation screen rather than a semiconductor direct-conversion detector, it illustrates the binder-dilution limit of solution-based routes. In 2022, Gimenez et al. embedded Gd_2_O_3_ particles into photoresist as a neutron conversion layer and coated it onto a field oxide field-effect transistor (FOXFET) for neutron-γ mixed-field dose measurements in BNCT [20]. In 2024, Wu et al. deposited ^10^B_4_C particles into 30 μm × 100 μm deep trenches on silicon wafers using a spin-coating method to fabricate a microstructured neutron detector. The feasibility of the device was preliminarily verified through I-V characteristic measurements and α-particle response tests [40].

#### 4.3.2. Impact on the Four Quality Dimensions of Conversion Layers

1. Areal Density (Moderate, Expected): The binder does not contribute to neutron capture capability but occupies volume, thereby reducing the effective nuclide areal density. In 2024, Wu et al. preliminarily confirmed the feasibility of fabricating detectors by spin-coating ^10^B_4_C into 30 μm × 100 μm trenches through I-V and α-response tests, but no absolute efficiency was reported [40]; the areal density achieved by solution routes therefore remains unquantified.
2. Density and Uniformity (Poor, Expected): The unavoidable pores between particles in solution-based films are the most severe performance-limiting factor. These pores reduce the effective density below the theoretical value, diluting the converter-isotope number density (and hence the macroscopic capture cross-section) per unit thickness, while the locally reduced stopping power actually lengthens the geometrical range of reaction products. The dominant penalty is thus the reduced capture probability at fixed thickness, compounded by binder dilution of the neutron-sensitive volume. Fedorov et al. reported that the sensitivity of the GYAGG/^6^LiF composite scintillation screen was comparable to that of the mature ZnS/^6^LiF screen, while its response speed was 40 times faster, indicating that solution-based methods have unique advantages in specific performance aspects (such as time response), despite the density limitation [39]. Currently, quantitative characterization data on the porosity of solution-based films are lacking in the neutron conversion layer literature, and this remains a key barrier to moving solution-based approaches from feasibility validation to performance optimization.
3. Adhesion/Interfacial Quality (Moderate, Expected): Adhesion depends on the chemical affinity between the binder and the substrate rather than the conversion material itself. Wu et al. (2024), Gimenez et al. (2022) and other groups have successfully fabricated functional detectors by solution-based methods, but none of these studies reported quantitative adhesion measurement data [20,39,40]. The adhesion can be optimized to some extent by selecting appropriate binder formulations; however, systematic studies in this aspect remain absent in the conversion-layer field.
4. Conformality (Moderate, Expected): The liquid-phase nature of solution-based methods allows the precursor suspension to fill high-aspect-ratio trenches by capillary and gravitational forces before drying, qualitatively indicating good trench-filling capability. No quantitative step-coverage ratio (t_bottom_/t_top_) has been reported for ^10^B_4_C or ^6^LiF composite films on detector-relevant substrates, and the subsequent drying step introduces porosity and binder-matrix shrinkage that complicate interpretation. The trench-filling route demonstrated by Wu et al. in 2024 [40] therefore represents the most realistic strength of solution-based methods in the conformality dimension even though it is not directly comparable to vapor-phase step-coverage data.

#### 4.3.3. Advantages and Limitations

Solution-based methods offer inherent compatibility with 3D microstructure filling, while requiring the lowest manufacturing cost and simplest equipment setup (ambient pressure and room temperature processing). Their fundamental limitation lies in the performance ceiling imposed by binder dilution and interparticle porosity, which is dictated by the physical nature of the solution-based process and cannot be completely eliminated through process optimization. The near-complete lack of quantitative thin-film quality metrics (such as porosity, density, and adhesion) is the main barrier between feasibility demonstration and performance optimization.

### 4.4. Chemical Vapor Deposition (CVD)

#### 4.4.1. Physical Mechanism

**Figure 8:**
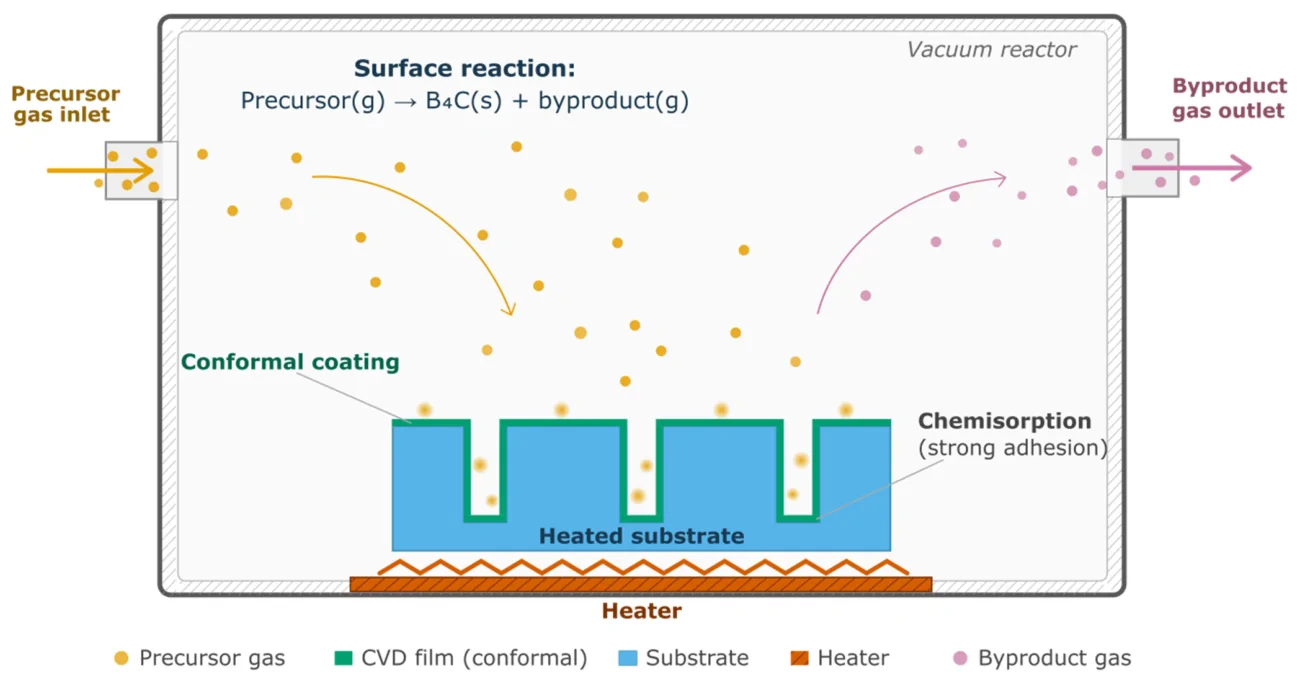
Schematic illustration of the chemical vapor deposition process. Gaseous precursors enter the vacuum reactor from the left inlet, diffuse to all surfaces of the 3D microstructures on the heated substrate, and undergo surface chemical reactions (Precursor(g) → B_4_C(s) + byproduct(g)). A conformal thin film is formed with uniform coverage of the top surface, sidewalls, and bottom surfaces, while gaseous byproducts are discharged through the outlet on the right side.

Chemical vapor deposition (CVD) uses gaseous precursors that undergo chemical reactions on heated substrate surfaces to form solid thin films. When the deposition rate is controlled by surface reaction kinetics (rather than precursor mass transport), precursor molecules have sufficient time to diffuse into the deep regions of high-aspect-ratio structures and react at the same rate on all accessible surfaces, enabling conformal growth. The chemisorption-based deposition nature provides the films with inherently strong adhesion to the substrate. A schematic illustration of the CVD process is shown in Figure 8.

Kim et al. achieved conformal growth of few-layer h-BN on Si-based nanogrooves with a high aspect ratio (~7:1) using pulsed-mode MOCVD. X-ray absorption fine structure spectroscopy and DFT calculations revealed the key mechanism: B–O bonds act as nucleation sites on non-catalytic SiO_2_ surfaces, promoting the formation of mixed sp_2_/sp_3_ hybridized BON_2_ and BN_3_ species during the initial stage of MOCVD precursor pulse injection, thereby enabling the conformal growth of few-layer sp_2_-h-BN with excellent step coverage [32].

In 2013, Huang et al. reported a honeycomb-structured Si diode (~2.8 μm holes, ~42 μm deep, aspect ratio > 12) in which LPCVD served for the in situ deposition of enriched boron (99.9% ^10^B), followed by diffusion at 800 °C to form a continuous p^+^–n junction and subsequent trench filling; the intrinsic thermal-neutron efficiency reached 26.1 ± 0.3% for a Maxwellian spectrum at normal incidence under zero bias (400 keV LLD), with a leakage current of ~2 × 10^−8^ A/cm^2^ [41]. In 2016, Wu et al. fabricated a microstructured thermal neutron detector by KOH anisotropic wet etching of (110) Si followed by LPCVD trench filling with enriched boron (99% ^10^B), reporting an intrinsic thermal-neutron detection efficiency of 16.3 ± 1.6% [42]. For CVD studies directly targeting neutron conversion layers, Choolakkal et al. achieved conformal ^10^B_4_C deposition in 8:1 aspect-ratio trenches using a triethylboron (TEB) single-source precursor in 2023, with near-unity step coverage (SC = 1) at ≤450 °C [43]; in a subsequent study, adding a competitive co-flow of Xe raised the step coverage from 0.71 to 0.97 in 10:1 aspect-ratio features [44]. In 2024, Choolakkal further deposited the films onto a prototype detector chip [45]. In 2012, Chaudhari et al. prepared ^10^B_4_C films using a hot-wire CVD route and performed neutron detection tests, one of the earliest studies to combine a CVD conversion layer with neutron detection experiments [46]. In 2021, Bhattarai investigated the feasibility of PECVD amorphous hydrogenated boron carbide (a-BC:H) for solid-state direct-conversion thermal neutron detection in a doctoral dissertation, providing another process route for CVD-based conversion layers [47].

#### 4.4.2. Impact on the Four Quality Dimensions of Conversion Layers

1. Areal Density (Not graded, Demonstrated): The deposition rate of boron-based films by CVD is relatively low, and the selection of precursors for neutron-sensitive materials (boron carbide and BN) is limited. Chaudhari et al. verified the basic feasibility of CVD ^10^B_4_C for neutron detection, but systematic efficiency optimization was not reported [46]. Choolakkal deposited the film onto a prototype detector chip, but direct tests of neutron detection efficiency have not yet been reported [45]. Device-level efficiency has been demonstrated in two LPCVD ^10^B studies that differ in microstructure geometry (16.3 ± 1.6% in KOH-etched trenches [42]; 26.1 ± 0.3% in a DRIE-defined honeycomb structure [41]); neither, however, reports the ^10^B areal loading achieved inside the microstructure or the trench filling fraction, so no areal-density grade is assigned (see note to Table 2). Deposition rates generally lower than those of PVD methods lengthen the processing time per unit thickness; they do not by themselves establish a ceiling on the attainable areal density, which remains unreported for CVD converters, so this dimension is not graded in Table 2.
2. Density and Uniformity (Moderate, Demonstrated): Reported densities of CVD boron carbide films are moderate (1.9–2.28 g/cm^3^ for B_5_C [43]), whereas under the surface reaction-controlled mode CVD achieves excellent conformal uniformity, which has been experimentally verified by the h-BN study of Kim et al. in 2023 (excellent step coverage in structures with an aspect ratio of ~7:1) [32] and by the ^10^B_4_C study of Choolakkal et al. in 2023 (near-unity step coverage, SC = 1, in 8:1 trench structures) [43], later improved to SC = 0.97 in 10:1 features by adding a Xe co-flow [44]. The conformality advantage of CVD over PVD in 3D microstructures is its defining physical characteristic.
3. Adhesion/Interfacial Quality (Good, Expected): The chemisorption-based nature of CVD provides inherently strong adhesion between CVD films and substrates. However, no studies have directly measured the adhesion strength of CVD conversion layers on semiconductor substrates or established its correlation with detector performance.
4. Conformality (Excellent, Demonstrated): CVD is currently the deposition technology with the strongest experimental conformality record for neutron-converter materials. Choolakkal et al. achieved near-unity step coverage (SC = 1) of ^10^B_4_C in 8:1 aspect-ratio trenches using a TEB single-source precursor at ≤ 450 C [43]; in a subsequent study, the same group raised the step coverage from 0.71 to 0.97 in 10:1 aspect-ratio features by adding a competitive Xe co-flow [44]. These bottom step-coverage values of 1.0 and 0.97 at 8:1 and 10:1, together with the few-layer h-BN result of Kim et al. in 7:1 Si nanogrooves [32], place CVD above the SC ≥ 0.9 threshold of the Excellent tier defined in the note to Table 2 and make it the most promising route for coating 3D microstructured detectors at the time of writing.

#### 4.4.3. Advantages and Limitations

CVD is currently the strongest candidate for conformal coating of 3D microstructured neutron detectors. Its near-unity step coverage in high-aspect-ratio trenches has been experimentally verified [43,44]. The current major limitations are that experimental validation of neutron detection efficiency rests on a small number of LPCVD ^10^B trench-filling reports (16.3 ± 1.6% [42]; 26.1 ± 0.3% [41]), all from the same chemistry, with device-level studies on other CVD chemistries still needed, as well as the relatively low deposition rate, which raises the processing time per unit thickness rather than defining a demonstrated areal-density ceiling.

### 4.5. Atomic Layer Deposition (ALD)

#### 4.5.1. Physical Mechanism

**Figure 9:**
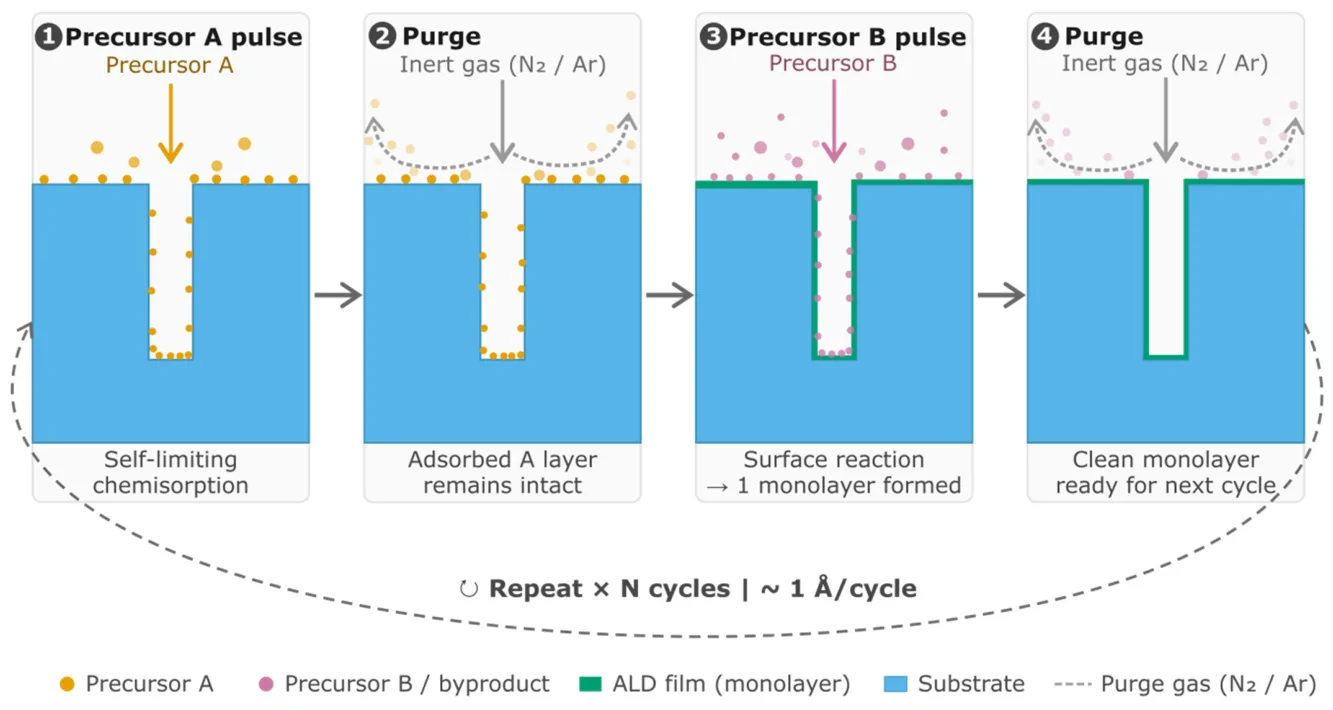
Schematic illustration of the atomic layer deposition process. Two precursors are alternately pulsed for deposition, and each cycle consists of four steps: (①) precursor A pulse, where molecules are self-limited chemisorbed onto all surfaces of the high-aspect-ratio trenches; (②) inert gas purge removes excess precursor A while maintaining the adsorbed layer unchanged; (③) precursor B pulse, where surface reactions occur with the adsorbed precursor A to generate an atomic-layer film and release byproducts; (④) purge of byproducts, producing a clean monolayer for the next cycle. Approximately 1 Å is deposited per cycle, and repeated N cycles enable layer-by-layer controllable growth.

Atomic layer deposition (ALD) achieves layer-by-layer atomic growth through alternating pulses of two or more precursors and self-limiting surface chemisorption reactions. A schematic illustration of the ALD process is shown in Figure 9.

Zhang J. et al. described the fundamental principles of ALD in their review: each precursor pulse chemically adsorbs onto all available surface sites until saturation, and the subsequent purge removes excess precursor before introducing the next reactant pulse, ensuring that only one (sub) monolayer is grown in each cycle [48]. This self-limiting mechanism provides ALD with three defining advantages: atomic-scale thickness control accuracy, pinhole-free films with near-theoretical density, and excellent conformality in structures with extreme aspect ratios. Chen et al. developed a multiscale CFD model of ALD in high-aspect-ratio nanostructures and experimentally verified conformal coating in nanopores with aspect ratios up to 100:1 [49].

ALD experimental studies specifically targeting neutron conversion layers remain scarce, and none identified in this survey reports device-level neutron detection performance.

#### 4.5.2. Impact on the Four Quality Dimensions of Conversion Layers

1. Areal Density (Not graded, Expected): The typical deposition rate of ALD is extremely low (~1 Å/cycle), so that a 1 μm-thick ^10^B_4_C film requires approximately ^10^4 cycles. This is a processing-time and cost penalty rather than a demonstrated limit on the thickness or isotope areal density that can be achieved, and no ALD conversion-layer study identified in this survey reports an areal density; no grade is therefore assigned, and the cell is marked Not graded in Table 2.
2. Density and Uniformity (Good, Expected): ALD films can approach theoretical density due to the layer-by-layer chemical adsorption mechanism, and their conformality in structures with aspect ratios exceeding 100:1 has been experimentally verified. These characteristics are conceptually consistent with the requirements of 3D microstructured neutron detectors; however, they have not been experimentally validated in this application scenario.
3. Adhesion/Interfacial Quality (Good, Expected): The self-limiting growth mechanism of chemisorption provides excellent interfacial quality and adhesion in principle, but experimental data on the adhesion of ALD films on semiconductor neutron detector substrates remain unavailable.
4. Conformality (Excellent, Expected): The self-limiting, surface-reaction-controlled growth mechanism of ALD provides, in principle, the highest intrinsic conformality among the five deposition technologies considered. Chen et al. experimentally verified conformal ALD coatings in nanopores with aspect ratios up to 100:1, with a multiscale CFD model extending the analysis to higher AR structures [49], and the underlying chemisorption mechanism does not impose a fundamental upper AR limit on principle.

#### 4.5.3. Advantages and Limitations

ALD combines the advantages of high thickness precision, dense thin films, and excellent conformality, making it suitable for the fabrication of 3D microstructured neutron detectors. However, the absence of ALD experimental studies on neutron conversion layers, together with the extremely uneconomical number of cycles required for thick-film deposition, means that the most realistic role of ALD in the foreseeable future is as an interface engineering tool. Specifically, ALD can be used to deposit ultrathin (~nm) adhesion layers or diffusion barrier layers (such as Al_2_O_3_) on semiconductor surfaces, using its atomic-scale interface control capability to provide an optimized interfacial foundation for subsequent thick-film deposition (sputtering or evaporation), though this concept also remains to be experimentally validated.

### 4.6. Comparison of Deposition Technologies and Thin-Film Quality

Based on the above analysis, Table 2 provides a qualitative comparison of five deposition technologies within the four-dimensional film quality framework. The evaluations in the table are judgments of process capabilities based on available published literature evidence, not universal absolute rankings. Optimization of specific process parameters can significantly alter the performance of individual dimensions.

**Table 2:** Qualitative comparison of deposition technologies and thin-film quality.

Deposition Technology	Areal Density	Density and Uniformity	Adhesion/Interfacial Quality	Conformality/Step Coverage	References
Magnetron sputtering	Excellent, Demonstrated: ^10^B ≈ 5.6 × 10^2^ μg/cm^2^ (3 μm × 2.39 g/cm^3^ × 0.777 ^10^B mass fraction for B/C = 5.18 at 96% ^10^B [28]); reference ≈ 5.6 × 10^2^ μg/cm^2^ (elemental ^10^B, 2.5 μm optimum, 2.34 g/cm^3^ [24], at the 96% enrichment of the anchor) → ≈100%	Good, Demonstrated: 2.39 g/cm^3^ (95% bulk); 2.2–2.3 g/cm^3^	Good, Demonstrated: cross-cut grade 5 on almost all Si, Al, Ti, and Cu samples; <5 only for stacks containing a Ti or Cu buffer layer [37]	Poor, Demonstrated: SC 0.23–0.27 at trench bottom/0.60–0.70 on slope (2.1 mm-deep grooves, 45° opening, AR ≈ 2:1); rating is per bottom SC [34]	[28,34,35,36,37,38]
Vacuum evaporation (thermal/electron-beam)	Excellent, Demonstrated: ^6^Li ≈ 1.45 × 10^3^ μg/cm^2^ (25 μm × 2.54 g/cm^3^ × 0.228 ^6^Li mass fraction at 95% ^6^Li, electron-beam evaporation [27]); reference ≈ 1.45 × 10^3^ μg/cm^2^ (^6^LiF at its 25 μm optimum, at the anchor density and 95% enrichment) → ≈100%; secondary anchors in the table note	Moderate, Expected	Good, Expected: bonding dominated by van der Waals forces; qualitative “quite good” adhesion on Si and other substrates, but no quantitative test [12]	Poor, Expected: SC ≈ 0 (line-of-sight, no sidewall data)	[12,25,26,27,37]
Solution-based methods	Moderate, Expected	Poor, Expected	Moderate, Expected	Moderate, Expected: liquid-phase trench filling qualitatively demonstrated, but no SC measured	[20,39,40]
CVD	Not graded, Demonstrated: device-level efficiency demonstrated in two LPCVD ^10^B studies (16.3 ± 1.6% [42]; 26.1 ± 0.3% [41]); neither reports the ^10^B areal loading inside the microstructure, so no grade is assigned	Moderate, Demonstrated: 1.9–2.28 g/cm^3^ (B_5_C, XRR, T-dep.)	Good, Expected: chemisorption-based growth; no measured adhesion for CVD converter layers	Excellent, Demonstrated: SC = 1.0 at AR 8:1 [43], improved from 0.71 to 0.97 at AR 10:1 by adding Xe co-flow [44]	[32,41,42,43,44,45,46,47]
ALD	Not graded, Expected: no ALD converter reports an isotope areal density; ~1 Å/cycle is a time penalty, not a ceiling	Good, Expected: near-bulk theoretical density in ALD process class, but no conversion-layer measurement	Good, Expected: chemisorption mechanism suggests strong adhesion in principle, but no quantitative data for neutron converter layers	Excellent, Expected: Chen verified ALD coatings in nanopores AR 100:1, but no conversion-layer experimental verification	[48,49]

Each cell reads <Rating>, <Status>: <anchor>, where Status is Demonstrated (rating supported by data reported in the cited references) or Expected (inferred from mechanism or analogous systems; the rating is then a qualitative tier, not a measured threshold). Cells marked Expected without a published anchor are qualitative tiers inferred from mechanism; no quantitative value is available in the literature. Ratings follow working conventions adopted in this review, evaluated at the thickness or aspect ratio stated in each anchor: areal density, as a fraction of the self-absorption-optimal areal density (Excellent ≥ 90% > Good ≥ 70% > Moderate ≥ 40% > Poor); density, as a fraction of bulk density from XRR/GIXRR or RBS (Excellent > 95% > Good ≥ 85% > Moderate ≥ 70% > Poor); adhesion, on the 0–5 residual-area cross-cut scale of [37] (5 = coating fully retained): Excellent, grade 5 throughout; Good, grade 5 except for isolated layer stacks; Moderate, 3–4; Poor, ≤ 2; and conformality, from the bottom-of-feature SC = t_bottom_/t_top_ [44], which is aspect-ratio dependent (Excellent ≥ 0.9 > Good ≥ 0.7 > Moderate ≥ 0.5 > Poor). For entries that rely on filling a 3D microstructure rather than on a planar coating, the planar self-absorption optimum does not apply; where the isotope loading inside the microstructure is not reported, the cell is marked Not graded. Reference value for the areal-density column: the isotope areal density at the self-absorption optimum reported for that material, evaluated at the isotope enrichment and film density of the anchor so that the two are compared like for like, namely elemental ^10^B at 2.5 μm, 2.34 g/cm^3^ and 96% enrichment for boron-based converters [24], and ^6^LiF at 25 μm, 2.54 g/cm^3^ and 95% enrichment for lithium-based converters [12,24]. Using instead the ^6^LiF density of 2.64 g/cm^3^ assumed in [24] would give 1.51 × 10^3^ μg/cm^2^ and a ratio of ≈96%, leaving every rating unchanged. Not graded = the cited work does not report the isotope areal density, or the reference value, needed for grading; a low deposition rate is read as a processing-time penalty, not as evidence of a loading ceiling. Secondary anchors for vacuum evaporation: 16.87 ± 0.37 μm ^6^LiF [12] → ^6^Li ≈ 9.8 × 10^2^ μg/cm^2^ (68% of the reference value); 5 μm ^6^LiF/diamond [26] → ^6^Li ≈ 2.9 × 10^2^ μg/cm^2^ (20%).

The matching recommendations between these deposition technologies and specific detector architectures are further elaborated in Section 5.5.

## 5. Microstructural Optimization and Architecture Adaptation

**Figure 10:**
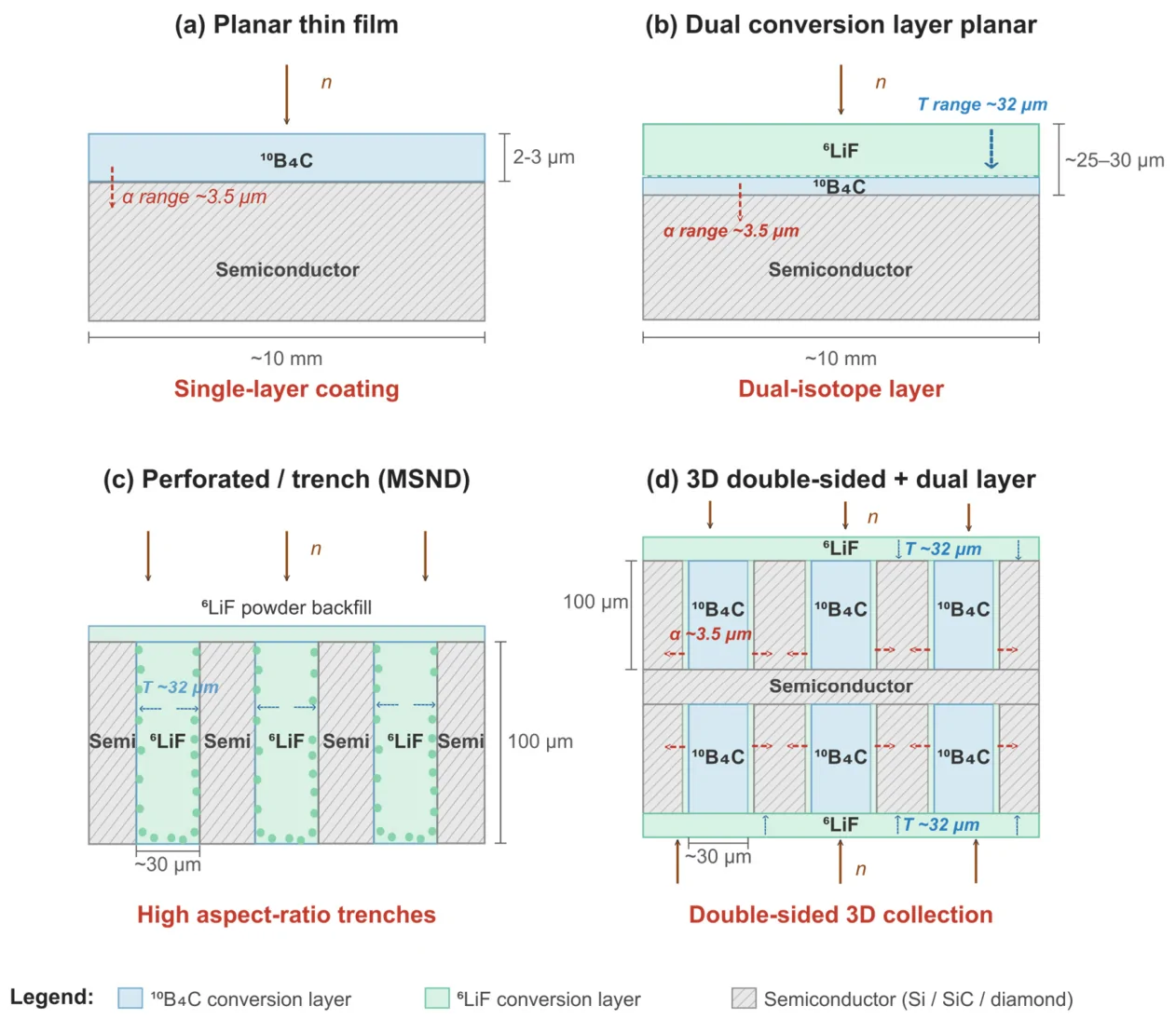
Structural evolution of semiconductor neutron detectors with conversion layers: (**a**) planar single-layer coating; (**b**) dual-isotope layer; (**c**) high-aspect-ratio trenches; (**d**) double-sided 3D charge collection structure.

Section 4 discussed the influence of different deposition technologies on the quality of conversion-layer films. Based on this analysis, this section explores five optimization strategies beyond simple single-layer coating, with a focus on their physical mechanisms, experimental validation status, and applicable conditions. The structural evolution of semiconductor neutron detectors with conversion layers is shown in Figure 10.

### 5.1. Isotopic Enrichment

The use of isotopically enriched materials (such as 95–99% ^10^B or 95% ^6^Li) instead of materials with natural isotopic abundance can increase the areal density by 4–5 times (^10^B) or approximately 13 times (^6^Li) at the same geometrical thickness, making it the most direct and effective optimization strategy. This approach has been validated by multiple independent laboratories: Zhu et al. used enriched ^10^B targets in 2023 [28], Hong et al. used 95% ^6^Li in 2022 [25], Wu et al. used 99% ^10^B in 2016 [42], and Hutchins et al. used 95% ^6^LiF particles in 2023 [13]. These studies define the best-supported optimization pathway. The major drawback is the substantially increased material cost (the price of enriched ^6^LiF can be tens to hundreds of times higher than that of natural LiF), but planar detectors built from natural-abundance materials typically show efficiencies below 1–2%, making isotopic enrichment a precondition for high-efficiency detection.

### 5.2. Thickness Optimization of Conversion Layers

Planar thin-film conversion layers exhibit an optimal thickness determined by the self-absorption effect. For single-sided, single-layer planar converters under perpendicular thermal-neutron (0.0253 eV) irradiation with a 300 keV LLD, Geant4 simulations place the intrinsic efficiency ceiling at ~4–5% (elemental ^10^B ~3.9% at 2.5 μm in that simulation; ^6^LiF ~4.3% at 25 μm) [24], consistent with the analytical results of McGregor et al. [7]. For ^10^B_4_C the optimum is quoted as a range, ≈2–3 μm (Section 2.4), rather than as a single thickness: carbon lowers the boron content per unit thickness while adding stopping power of its own, and the spread between studies reflects their different simulation conditions. A stoichiometric estimate that reproduces the ^10^B areal density of the elemental-boron optimum falls near the upper end of this range, and the Table 2 reference is expressed as an areal density rather than as a thickness precisely so that this spread does not propagate into the rating. This ceiling is not universal: it rises with a lower LLD, with back-irradiation or double-sided geometries, and with dual-isotope coatings (e.g., 6.78% for a planar ^6^LiF + ^10^B coating [24]; 13.5% for a ^6^LiF/^10^B sandwich [7]). Both planar and three-dimensional routes therefore move beyond this ceiling: within planar architectures, the measures listed above raise it to 6.78% and 13.5% with no three-dimensional structuring at all, whereas three-dimensional microstructures relax the self-absorption constraint itself by shortening the transport distance to the semiconductor (Section 5.4) and reach the highest reported intrinsic efficiencies. This ceiling should consequently be read as a property of one specific configuration rather than as a bound on planar detectors in general.

### 5.3. Dual Conversion Layers and Multilayer Film Structures

The dual conversion-layer strategy improves overall efficiency by spatially combining different isotopes (such as ^6^LiF + ^10^B_4_C), exploiting their complementary neutron cross-section-energy dependence and reaction-product energy distributions. Wang, H. et al. performed Geant4 simulations of diamond thermal neutron detectors and showed that dual conversion layers could increase the efficiency of planar structures from 4.4% to 7.2%, while the efficiency could exceed 70% in double-sided dual-conversion-layer 3D trench structures (with a trench depth of 100 μm) [50]. Zhang, L. et al. demonstrated through simulation studies in 2021 and 2023 that dual conversion layers combined with dual-trench structures significantly outperform single conversion-layer configurations, and that the substantially increased counting efficiency of dual trenches in the low-energy region is the fundamental physical reason for the efficiency improvement [24,51]. Nan et al. proposed a stacked microstructure approach that provides a pathway toward scalable fabrication by accumulating efficiency through stacking standardized single-layer detector units rather than pursuing extreme structural complexity in a single device [52].

The efficiency improvements of dual conversion layers/multilayer films currently come mainly from Geant4/MCNP simulation predictions; experimental validation remains to be conducted.

### 5.4. Three-Dimensional Microstructures

The concept of microstructured semiconductor neutron detectors (MSNDs) was first proposed and experimentally validated by McGregor et al. in 2009 [53]. The fundamental idea is to etch deep holes or trench arrays into semiconductor diodes and refill them with neutron conversion materials, enabling the secondary charged particles generated by neutron capture to enter adjacent semiconductor fins within a short distance (shorter than the particle range) and generate signals, thereby substantially reducing self-absorption losses [53]. Early Monte Carlo modeling by Shultis and McGregor in 2006 theoretically predicted that this microstructured design could achieve an order-of-magnitude improvement in intrinsic efficiency [23]. Hutchins et al. reported representative MSND performance: the average intrinsic thermal neutron detection efficiency before irradiation reached 24.09 ± 0.35%, well above the ~4–5% ceiling computed for an enriched, single-sided, single-layer planar converter under the same 300 keV lower-level discriminator (Section 5.2) [13,24]. Wu et al. fabricated a microstructured detector on (110) silicon wafers using a low-cost KOH anisotropic wet etching process, achieving an intrinsic thermal-neutron detection efficiency of 16.3 ± 1.6% under self-powered (0 V) operation, with a leakage current of ~ 6.7 × 10^−6^ A/cm^2^ at −1 V [42]. Tan et al. fabricated and investigated a ^6^LiF-based deep-trench Schottky-type SiC neutron detector, in which a SiO_2_ dielectric layer was formed through thermal oxidation to suppress leakage current, achieving an efficiency of 6.57% [54].

When the detector architecture transitions from planar to 3D structures, the key challenge for conversion-layer deposition shifts from large-area uniformity to conformal coating. At this dimension, the capability differences among deposition technologies are most pronounced: CVD and ALD are inherently suitable for conformal coating due to their surface reaction-controlled mechanisms; the line-of-sight effect of PVD results in much lower film thickness at trench bottoms and sidewalls compared with the top surface; the fluid infiltration capability of solution-based methods provides unique advantages for trench filling, but shrinkage and pore formation during drying introduce new challenges. Currently, all experimental MSNDs are fabricated by deep reactive-ion etching (DRIE) of Si (or, in low-cost variants, KOH anisotropic wet etching) followed by mechanical backfilling of enriched ^6^LiF microparticulate powder or filling with enriched ^10^B [13,41,42,53], while CVD filling with enriched ^10^B by LPCVD has already been validated at device level in two studies differing in microstructure geometry (16.3 ± 1.6% in KOH-etched trenches [42]; 26.1 ± 0.3% in a DRIE-defined honeycomb structure [41]), the performance of ALD-coated 3D microstructures remains to be validated. In addition, Kolesnikov et al. explored a ^10^B-based macrostructured cathode approach in 2025 (a gas-detector cathode operating with a working gas of CF_4_/C_4_H_10_ = 80/20, cited here solely for its sputtered ^10^B_4_C process data, as its detection mechanism differs from semiconductor direct conversion), providing an alternative perspective for conversion-layer structural design [55].

### 5.5. Matching Between Deposition Technologies and Detector Architectures

Large-area planar detectors (imaging screens and large-area monitors) require high thickness uniformity over active areas ranging from hundreds of square centimeters to square-meter scale, while maintaining sufficient areal density to support effective neutron capture. Magnetron sputtering is currently the most strongly supported matching deposition technology in terms of both uniformity and areal density. Vacuum evaporation is competitive in areal density, but its large-area uniformity is limited by the geometry of the evaporation source. For CVD the isotope areal density delivered inside microstructures has not been reported, so it cannot yet be ranked on this dimension (Table 2); for solution-based methods, binder dilution and interparticle porosity lower the attainable areal density, which is currently insufficient for competition with PVD.

For 3D microstructured detectors (trench-type and perforated structures), the core requirement shifts from large-area uniformity to conformal coating within high-aspect-ratio structures. CVD has an inherent advantage in conformality due to its surface reaction kinetics-controlled deposition mechanism. Conformal deposition has already been demonstrated in trenches with aspect ratios of 8:1–10:1, making it the deposition technology currently closest to this requirement. The liquid-phase infiltration capability of solution-based methods provides a low-cost filling pathway, but the issues of pore formation and shrinkage during drying remain unresolved. The line-of-sight effect of PVD results in the poorest conformal capability, and current MSNDs therefore rely on trenches defined by DRIE or, in low-cost variants, by KOH anisotropic wet etching, and either backfilled with enriched ^6^LiF microparticulate powder [13,53] or filled with enriched ^10^B by LPCVD [41,42], rather than on direct line-of-sight coating.

Flexible neutron detectors (wearable dosimeters and curved detectors) require bending compatibility and low-cost large-area fabrication. Solution-based methods are naturally compatible with roll-to-roll continuous manufacturing and represent the most reasonable choice for this scenario. However, the quantitative effects of binder dilution and interparticle porosity on the upper efficiency limit remain unclear. PVD and CVD films generally exhibit relatively high residual stress and are prone to cracking under bending strain unless specialized stress-engineering strategies are employed.

High-temperature and high-radiation environments (online reactor monitoring and fusion diagnostics) impose dual challenges of interfacial stability and radiation resistance, which together lead to interface degradation and dead-layer thickening. Experimental evidence in this direction is currently very limited. Systematic comparisons across materials, deposition technologies, and substrates under high-temperature and high-radiation conditions remain largely unexplored and represent an urgent research direction.

## 6. Interface Engineering

The conversion layer-semiconductor interface is a critical region that determines detector performance. Reaction products lose energy when traversing this dead-layer region, which is typically tens to a few hundred nanometers thick (heavily doped contact/window layers), although partially depleted structures may exhibit thicker inactive regions, while interfacial defect states affect charge collection efficiency, and residual stress influences long-term reliability. Yet compared with areal density and density/uniformity, the interfacial dimension is the least studied in the literature.

### 6.1. Dead Layer Effect

**Figure 11:**
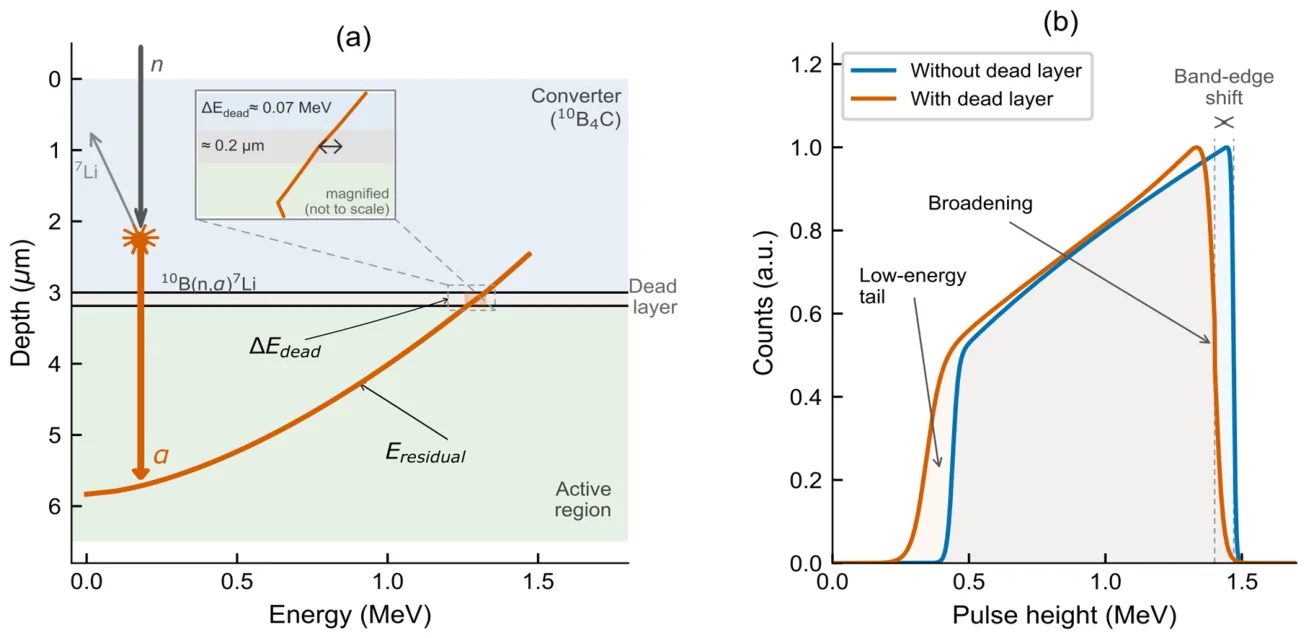
Dead layer effect at the conversion layer/semiconductor interface. (**a**) Schematic illustration of α-particle energy loss in the dead layer: the energy loss ΔE_dead_ during the traversal of the interfacial dead layer before entering the active region and the remaining energy E_residual_; (**b**) Schematic pulse height spectra based on the energy loss model: because reaction products are generated over continuous distributions of depth and emission angle in a finite-thickness converter, the spectrum is a continuum bounded by a band edge rather than a resolvable peak. Compared with the ideal case without a dead layer, the spectrum exhibits band-edge shift, broadening, and low-energy tailing.

The “dead layer” refers to a region within a certain depth beneath the semiconductor surface that cannot effectively collect charges due to dopant distribution, surface states, or lattice damage [7]. In semiconductor radiation detectors, the presence of a dead layer indicates that the energy lost by reaction products when traversing this region does not contribute to the output signal, which is equivalent to introducing an “inactive absorber” layer between the conversion layer and the semiconductor sensitive region. The interfacial dead layer between the conversion layer and semiconductor causes charged particles to lose part of their energy before entering the active region, as illustrated in Figure 11.

The physical origins of the dead layer include: (1) the non-depleted doped layer at the semiconductor surface: for P–N junction-based detectors, the electric field within the heavily doped contact layer is zero or extremely weak, resulting in a charge collection efficiency approaching zero; (2) surface state density (Dit)-induced Fermi-level pinning, which forms depletion or accumulation layers at the surface and modifies the boundary of the effective sensitive region; and (3) deep-level traps introduced by lattice damage during mechanical processing or deposition processes [10].

For semiconductor detectors coated with conversion layers, the deposition process itself (high-energy particle bombardment, elevated temperature, and chemical reactions) may introduce additional defects into the semiconductor surface, thereby thickening or generating new dead layers. In 2023, Zhang, C. et al. reported that the indirectly coupled configuration (depositing ^10^B_4_C on an aluminum substrate followed by bonding with a silicon detector) outperformed the directly deposited configuration [38]. This result strongly suggests that direct deposition of conversion layers onto semiconductor surfaces may introduce adverse interfacial effects, although the underlying physical mechanisms (sputtering-induced damage, interfacial oxidation, and thermal stress) have not yet been independently resolved. In 2026, Tan et al. characterized deep-level defects in 4H-SiC using Deep Level Transient Spectroscopy (DLTS), identifying three major trap states; however, the investigated interface was a thermal oxide interface rather than the conversion layer–semiconductor interface itself [54].

The current literature lacks experimental investigations directly targeting the dead layer thickness, interfacial trap density, and their quantitative impacts on charge collection efficiency at the conversion layer–semiconductor interface. The superior performance of indirect coupling over direct deposition reported by Zhang, C. et al. [38] and the DLTS characterization of thermal oxide interfaces by Tan et al. [54] provide indirect evidence of interfacial effects and methodological references for characterization, respectively; however, neither study directly addresses the influence of conversion layer deposition itself on dead layer formation. Until this gap is closed, interfacial quality cannot enter process selection as a quantitative criterion.

### 6.2. Stress and Adhesion

Thin film stress is a fundamental concept in thin film engineering and is generally decomposed into two components: intrinsic stress and thermal stress [56]. Intrinsic stress originates from the non-equilibrium microstructure formed during thin film growth, where atoms are “frozen” at non-equilibrium positions during deposition, causing the film to exist in a compressive or tensile state relative to its free state. According to Thornton’s Structure Zone Model (SZM) [57], films deposited at low temperatures (Ts/Tm < 0.3) tend to form porous columnar structures, where atomic interactions at grain boundaries can generate tensile stress; in contrast, high-energy particle bombardment (such as the atomic peening effect during sputtering) tends to introduce compressive stress. Thermal stress arises from the mismatch in the coefficient of thermal expansion (CTE) between the film and substrate, where different contraction behaviors during cooling from the deposition temperature to room temperature generate thermoelastic stress at the interface.

For neutron conversion layer systems, Lai et al. [37] measured the adhesion of ^10^B_4_C on Al, Ti, Cu, and Si substrates using a cross-cut tape test on the same 0–5 residual-area scale defined in Section 4.1.2. No direct experimental correlation has yet been established between adhesion and neutron detection efficiency. Adhesion evaluation and neutron efficiency measurements have never been jointly performed on the same device, preventing quantitative determination of the relationship between adhesion level and device performance.

For strategies to improve interfacial quality, Das et al. investigated the passivation effect of N_2_ annealing on interface traps in 4H-SiC/SiO_2_ systems in 2023 [58], while Wang, R. et al. reported the influence of interfacial boron concentration on trap passivation in 2024 [59]. Although these studies were not conducted specifically for neutron conversion layers, the revealed principles of interface passivation provide direct references for conversion layer-semiconductor interfaces. A conceptually reasonable but experimentally unverified approach is to first deposit an ultrathin buffer layer (such as Al_2_O_3_, approximately 5–10 nm) on the semiconductor surface via ALD, using the atomic-level interface control capability of ALD to simultaneously achieve stress relaxation (through CTE gradient transition) and interfacial defect passivation (through chemical passivation of dangling bonds), followed by thick-film deposition of the conversion layer.

### 6.3. Radiation Damage and Interfacial Degradation

Radiation-induced damage is a critical factor limiting the long-term reliability of neutron detectors, with its physical mechanisms involving multiple aspects. From a dosimetric perspective, non-ionizing energy loss (NIEL) is a fundamental parameter for quantifying displacement damage. It represents the fraction of energy transferred from incident particles to atomic nuclei, causing atoms to be displaced from their lattice sites, and is typically expressed in units of MeV·cm^2^/g. In their 2013 review, Srour and Palko described the physical mechanisms of displacement damage in semiconductor devices, including the generation kinetics of defect clusters and isolated defects, the energy and particle-type dependence of NIEL, carrier concentration variation effects, and random telegraph signal (RTS) noise, the basis for understanding radiation-induced degradation of detector performance [60]. The cumulative effects of displacement damage can be quantified by dpa (displacements per atom), which is proportional to the product of particle fluence and NIEL.

For neutron conversion layer detectors, the sources of radiation damage are dual: (1) direct irradiation from external neutron fields; and (2) recoil implantation induced by nuclear reaction products generated within the conversion layer itself.

The irradiation durability study of MSNDs by Hutchins et al. in 2023 is among the few studies providing quantitative data on conversion layer interfacial degradation [13]. As the thermal neutron fluence increased from 10^7^ to 10^12^n/cm^2^, the leakage current increased from <11 nA to approximately 300 nA (−3 V), while the efficiency decreased from 24.09% to 4.42%. The performance degradation was attributed to displacement damage induced by reaction products within the p-n diode.

This result reveals a frequently overlooked design constraint: the nuclear reaction products generated by the conversion layer not only contribute to signal generation but also continuously irradiate the semiconductor material of the detector itself. Therefore, the design of highly durable conversion layers should consider not only external neutron irradiation but also the cumulative damage caused by continuous bombardment from internal nuclear reaction products on the interface. However, quantitative irradiation durability data for such MSNDs remain sparse, with Hutchins (2023) [13] a rare example, and cross-material, cross-process, and cross-substrate comparisons of irradiation durability remain unavailable.

## 7. Computational Simulation Methods

Computational simulation is indispensable in neutron conversion layer design: it enables rapid exploration of multidimensional parameter spaces and identification of optimal design regimes without the need for costly experiments. This section briefly reviews three mainstream simulation tools and their methodological roles in conversion layer optimization.

### 7.1. Geant4 Monte Carlo Simulation

Geant4 is currently the most widely adopted Monte Carlo toolkit for semiconductor neutron detector simulations. Its core capabilities include a comprehensive neutron–nuclear reaction cross-section database (NeutronHP), full particle transport chain simulations from incident neutrons to secondary charged particles, and compatibility with CAD model import [61]. Multiple independent simulations have consistently predicted the optimal thickness range of planar thin films and the efficiency enhancement enabled by 3D structures. Furthermore, the good agreement between the experimental efficiency reported by Zhu et al. in 2023 (3.3 ± 0.3%, measured on a ceramic GEM gas detector cathode) and the Geant4 simulated value (3.4%) cross-validates Geant4 for this field [28].

Representative simulation findings include the optimization of SiC trench detectors by Zhang, L. et al. in 2021 and 2023, involving the transition from single-layer to double-trench dual-conversion-layer configurations [24,51]; the Geant4 simulation of a diamond double-sided dual-conversion-layer 3D detector by Wang, H. et al. in 2025, predicting an efficiency exceeding 70% [50]; the stacked-structure approach proposed by Nan et al. in 2025 for doubling detection efficiency [52]; and the optimization of conversion layer materials, thicknesses, and electrode parameters for diamond detectors conducted by Lin et al. in 2024 [22]. Li et al. developed a complete simulation package for self-powered neutron detectors (SPNDs) based on Geant4, and its methodology also provides valuable references for the simulation of conversion layer detectors [62].

### 7.2. SRIM Ion Transport Simulation

SRIM (Stopping and Range of Ions in Matter) is based on the binary collision approximation (BCA) and provides quantitative data on the energy loss (dE/dx), range, and range straggling of α, ^7^Li, and ^3^H particles in conversion layer materials. Xiang et al. employed SRIM in their multiscale simulation framework for GaN neutron detectors to determine the maximum ranges of secondary charged particles, which were used as geometric parameters for subsequent Geant4 and TCAD simulations [63].

A key application of SRIM in conversion layer design is the quantification of self-absorption effects. For a given film thickness d, SRIM can calculate the probability that reaction products generated at a depth x can reach the semiconductor, providing the microscopic basis for full-scale Geant4 simulations.

### 7.3. TCAD and Multiphysics Coupled Simulation

TCAD converts the energy deposition distributions obtained from Monte Carlo simulations into actual electrical signal responses. The MC-TCAD coupled simulation of GaN p-i-n detectors reported by Xiang et al. in 2025 is a state-of-the-art implementation in this field [63]. Key findings include that a 0.4 MeV γ-ray energy threshold can effectively suppress γ interference; low-work-function metals (such as Al) forming ohmic contacts can significantly enhance signal collection efficiency; and increased trap concentrations simultaneously increase reverse leakage current and deteriorate transient response. The application of TCAD in the conversion layer field remains limited to a small number of studies, and the trap parameters and interface state density models used in simulations have not yet been systematically calibrated against experimental data from neutron detectors.

The complete simulation workflow, from neutron source definition, through neutron transport and nuclear reactions in Geant4, SRIM-based energy loss analysis, and finally TCAD-based charge collection and pulse height spectrum simulation, has been theoretically established, but it still lacks experimental closed-loop validation.

## 8. Limitations and Future Perspectives

This review has summarized the current research status of neutron semiconductor detector conversion layers from six perspectives: nuclear reaction physics, material selection, deposition processes, microstructure, interface engineering, and computational simulation. Building on this analysis, this section identifies the challenges that remain and outlines future research directions.

### 8.1. Systematic Comparison of the Relationship Between Processing Conditions and Thin Film Quality

The comparison matrix presented in Section 4 of this review reveals a significant imbalance: existing thin film quality data are highly concentrated on the single system of magnetron-sputtered ^10^B_4_C, while key parameters such as Density and Uniformity and step coverage for CVD, ALD, and Solution-based methods lack systematic experimental reports. Moreover, comparative studies under identical substrate and material conditions across different deposition processes are nearly absent. Because of this gap, process selection still relies largely on empirical judgment rather than quantitative criteria.

Therefore, future research should focus on cross-process comparative studies, in which the same material is deposited on identical substrates using different methods and evaluated using unified characterization metrics. Such studies do not necessarily require new experimental facilities but demand more systematic approaches in terms of characterization dimensions. The efficiency values reported in the literature, together with the measurement conditions that make them comparable or not, are collected in Table 3.

### 8.2. Conformal Deposition on 3D Microstructures

3D microstructures provide a viable pathway to overcome the efficiency limitations of planar configurations, and conformal deposition is the core processing challenge. Currently, reported experimental MSNDs are fabricated by DRIE- or, in low-cost variants, KOH-defined trenches, subsequently backfilled with enriched ^6^LiF microparticulate powder or filled with enriched ^10^B by LPCVD [13,41,42,53]. Although the conformal coating capability of CVD and ALD in high-aspect-ratio structures has been preliminarily demonstrated [32,43], device-level efficiency has so far been validated only for LPCVD ^10^B trench filling, in studies such as those on KOH-etched trenches (16.3 ± 1.6% [42]) and DRIE-defined honeycomb structures (26.1 ± 0.3% [41]); validation on other CVD chemistries and on ALD-coated 3D microstructures is still missing.

ALD features high thickness precision, dense film formation, and excellent conformality, making it suitable for the fabrication of 3D microstructured neutron detectors, but its low deposition rate and cost limit its application in large-area devices. Achieving a balance among efficiency, cost, and scalability will be critical for advancing this approach toward practical implementation.

### 8.3. Emerging Material Systems and Functional Integration

Some emerging materials aim to integrate neutron conversion and charge collection functions within a single material system, of which h-BN is the most prominent example. Maity et al. achieved a normalized thermal neutron detection efficiency of 58.9 ± 2.4% using h-BN [31], although its performance is highly sensitive to material defects. Kargar et al. explored lithium-based semiconductor materials such as LiInSe_2_ [64], while simulations by Blevins and Yang indicated that direct ^10^B doping in Ga_2_O_3_ still exhibits lower efficiency than external conversion layer schemes [65]. Medic investigated amorphous boron carbide/amorphous silicon heterojunction devices [66]. All of these materials are intrinsic-conversion semiconductors and are discussed here only for cross-process comparison rather than as external conversion layers.

Perovskite materials have also demonstrated potential for fast neutron detection. El Bouanani et al. demonstrated the feasibility of coupling perovskite materials with ^10^B conversion layers for thermal neutron detection [67]. Gao et al. reported that the two-dimensional perovskite BDAPbBr_4_ exhibits a μτ product of 3.3 × 10^−4^ cm^2^/V [68], while PEA_2_PbBr_4_ thin films developed by Fratelli et al. enabled real-time monitoring of fast neutron flux [69], but radiation stability and material toxicity remain unresolved challenges.

In summary, functional integration advances only if material growth maintains semiconductor-grade lattice quality while incorporating neutron-sensitive elements. At present, this requirement remains difficult to achieve in the near term, and external conversion layer schemes are expected to remain the dominant approach.

### 8.4. Critical Assessment of Technology Maturity and Limiting Factors

The preceding subsections list what still needs to be done. This subsection instead records the authors’ own judgement of how the field is likely to develop over the next few years, with the reasoning behind it. It is deliberately written as a testable viewpoint rather than a consensus.

Consolidation is most likely for magnetron-sputtered ^10^B_4_C, on planar substrates first and on micromachined substrates increasingly. Two factors favour it: this material–process pair already has the largest evidence base reviewed here (Section 4.1 and Section 8.1), and the required tooling and ^10^B supply chain exist at industrial scale, as shown by 1500 × 400 mm^2^ coatings with better than ±3% thickness uniformity [28]. Its ceiling in the single-sided, single-layer planar configuration is set by physics rather than by film quality: self-absorption caps the intrinsic efficiency of that configuration near 4% (Section 5.2), so further process optimization there will yield diminishing returns. The larger remaining gain comes from architecture, that is, from shortening the reaction-product transport path by 3D microstructuring, although the planar measures listed in Section 5.2 (lower discrimination thresholds, back-irradiation, double-sided and dual-isotope geometries) also raise the ceiling, to 6.78% and 13.5%. Progress along that route is gated by micromachining and by sidewall and dead-layer quality at least as much as by conversion-layer chemistry.

^6^LiF will remain the reference material and the pragmatic planar option, because thermal evaporation of planar films is inexpensive and ≥95% enriched material is commercially available, but its lower cross section (940 barn, versus 3840 barn for ^10^B) and the ~32 μm tritium range keep the intrinsic efficiency near 4% in the single-sided, single-layer planar geometry at a 300 keV LLD (Section 5.2), a bound set by that configuration rather than by the material. We therefore expect it to persist as a benchmark and a low-cost option rather than to become a performance leader. The 3D route, by contrast, rests on a separate process chain: reported MSNDs are built by mechanical backfilling of enriched ^6^LiF microparticulate powder into DRIE- or KOH-defined trenches [13,53], or by LPCVD filling with enriched ^10^B [41,42], rather than by evaporation, whose line-of-sight transport cannot coat high-aspect-ratio features.

Conformal deposition techniques will remain conditional. ALD offers the best thickness control and conformality, yet its deposition rate and cost confine it to high-aspect-ratio structures where conformality is decisive; we do not expect it to displace sputtering for large-area devices within this horizon. Device-level demonstrations of CVD filling remain sparse; the two identified for LPCVD ^10^B trench filling [41,42] both use the same chemistry and differ only in microstructure geometry. Whether it becomes a mainstream 3D route depends on reproducing such results across chemistries and on continued access to enriched precursors.

We further judge Gd-based and solution-based layers, and intrinsic-conversion semiconductors, to remain niche in this period. Gd provides the largest cross sections (60,900 barn and 254,000 barn for ^155^Gd and ^157^Gd), but its conversion electrons are low in energy and short in range and are accompanied by a complex de-excitation cascade that complicates γ discrimination, while device-level evidence on semiconductor platforms remains sparse (Section 3.4); its advantage is therefore realized mainly in specialized configurations such as particle-loaded polymer layers. Solution-based layers are attractive where cost, flexible substrates, or very large areas dominate, but binder dilution and porosity cap the achievable areal density, so they will likely stay confined to applications that trade efficiency for coverage. h-BN shows the highest efficiency of any single material reviewed here (58.9 ± 2.4% [31]) and is the most credible route to genuine functional integration, yet its performance is governed by defect density rather than by nuclear physics; until epitaxial quality reproducibly reaches semiconductor grade, its near-term role will be in applications with less stringent efficiency requirements, such as neutron imaging. Perovskite-based schemes remain exploratory, with radiation stability and toxicity unresolved.

The limiting factor that we consider most often underestimated is neither a material nor a process but evidence. Reported efficiencies are rarely comparable because definitions, lower-level discriminators, and neutron fields differ between studies [15]; cross-process benchmarks are almost absent (Section 8.1); and quantitative irradiation-durability data exist for essentially one device set [13]. Until standardized benchmarks and reporting conventions are established, comparative claims will remain difficult to substantiate, and this, rather than any single technical bottleneck, is what most constrains rational process selection today. Two results would force us to revise the ranking above: a device-level demonstration of conformal ALD or CVD coating in 3D microstructures that sustains high efficiency and long-term stability, and a cross-process benchmark performed under identical substrate and characterization conditions.

## 9. Conclusions

This review has summarized neutron semiconductor detector conversion layers from six perspectives: nuclear reaction physics, material selection, deposition processes, microstructural optimization, interface engineering, and computational simulation. Based on four key dimensions, namely areal density, density and uniformity, conformality, and interfacial quality, the main conclusions are as follows:

At the material level, ^6^LiF is the most widely used benchmark material, featuring long reaction product ranges and good compatibility with planar fabrication processes; however, its performance is limited by the relatively low neutron absorption cross-section and larger optimal thickness. ^10^B_4_C exhibits a higher cross-section, thinner optimal thickness, and the richest thin film characterization data, making it the material system with the strongest evidence base for the relationship between processing conditions and thin film quality. h-BN integrates the dual functions of a conversion layer and semiconductor, but its material quality remains a major bottleneck. Gd and its oxides possess the highest neutron absorption cross-sections; however, the low energy and short range of conversion electrons limit their efficiency advantages in thin film configurations.

At the processing level, magnetron sputtering has the strongest evidence base for uniformity and areal density, making it the dominant choice for current large-area planar detectors. Vacuum evaporation exhibits competitive performance in terms of areal density, but large-area uniformity is limited by geometric constraints. CVD possesses inherent advantages in conformal coating and is one of the most promising processes for 3D microstructured detectors. ALD provides the highest precision in thickness control and conformality; however, its low deposition rate restricts its application in large-area devices. Solution-based methods offer the lowest cost and are suitable for flexible substrates, but binder dilution and porosity issues limit their achievable efficiency.

At the structural level, the efficiency of planar thin-film conversion layers is constrained by self-absorption effects. For a single-sided, single-layer planar converter under perpendicular thermal-neutron irradiation with a 300 keV lower-level discriminator, the optimal thicknesses of ^10^B_4_C and ^6^LiF are approximately 2–3 μm and 25–30 μm, respectively, corresponding to an intrinsic efficiency ceiling of approximately 4–5%. This ceiling is configuration-specific rather than universal: lower discriminator settings, back-irradiation or double-sided geometries, and dual-isotope or multilayer planar stacks raise it to 6.78% and 13.5% without any three-dimensional structuring. Three-dimensional microstructures provide a further and larger gain by shortening the transport distance of reaction products to the semiconductor, with MSNDs achieving intrinsic efficiencies exceeding 24%, while simulated efficiencies of dual-conversion-layer 3D structures can exceed 70%.

At the interface level, dead layer effects, residual stress, and radiation damage are critical factors affecting detector performance and long-term reliability; however, experimental data in this area remain extremely limited. Quantitative data on the irradiation durability of MSNDs remain sparse, with the work of Hutchins et al. [13] a rare example, while cross-material and cross-process comparisons of interfacial properties remain largely unexplored.

Research on conversion layers for neutron semiconductor detectors is currently transitioning from empirical process exploration toward rational thin film engineering. Advancing this transition requires sustained efforts in three key directions: establishing cross-process benchmark experiments for thin film quality comparison, promoting device-level validation of conformal deposition on 3D microstructures, and exploring practical pathways for functional integration of emerging material systems. As these areas mature, neutron semiconductor detectors can enter wider practical use in security inspection, fusion diagnostics, and radiation dose monitoring.

## Figures and Tables

**Table 1:** Key Nuclear Reaction Parameters of Candidate Neutron Conversion Reactions.

Nuclear Reaction	Thermal Neutron Cross Section, σ (Barn)	Q Value (MeV)	Main Reaction Products	Product Energy (MeV)	Typical Range (μm)
^10^B(n,α)^7^Li	3840	2.79 (ground state)/2.31 (excited state)	α+^7^Li(+γ)	α: 1.47–1.78, ^7^Li: 0.84–1.01	α: ~3.5 (in B_4_C) ^7^Li: ~1.6 (in B_4_C)
^6^Li(n,α)^3^H	940	4.78	α+^3^H	α: 2.05, ^3^H: 2.73	α: ~6–6.5 (in LiF) ^3^H: ~32 (in LiF)
^155^Gd(n,γ)	60,900	~8.54	Internal conversion electrons + γ	0.039–0.295 MeV (discrete lines)	Electrons: ~10–100
^157^Gd(n,γ)	254,000	~7.94	Internal conversion electrons + γ	0.029–0.276 MeV (discrete lines)	Electrons: ~10–100

**Table 3:** Quantitative comparison of reported neutron-detection efficiencies.

Ref.	Material/Process	Detector Type/Geometry	Enrichment/Thickness/Areal Density	Efficiency (Definition and Value)	Neutron Field/LLD
[12]	^6^LiF vacuum evaporation	Semiconductor/Planar	n.r.; 1.8/16.87 ± 0.37 μm (measured); 458/4290 ± 94 μg/cm^2^	not reported	Moderated AmBe (2.2 × 10^6^ n/s, ~2000 thermal n/cm^2^·s); LLD = software 1.8/1.5 MeV, hardware ~0.4 MeV
[13]	^6^LiF powder mechanical backfill into DRIE MSND	MSND/3D DRIE trenches	95% ^6^Li; n.r.; n.r.	intrinsic (pre-irradiation, −3 V): 24.09 ± 0.35%	KSU TRIGA thermal neutrons; LLD not reported
[17]	diamond/SiC/Ga_2_O_3_/GaN + B_4_C/LiF (Geant4 10.7)	Semiconductor/Planar thin-film coating	natural B/natural Li; B_4_C 2.6 μm @ 300 keV/1.7 μm @ 900 keV; LiF 30.6 μm @ 300 keV/27.0 μm @ 900 keV; n.r.	intrinsic (Geant4): absolute efficiency not given in text (only Figure 3/4 curves); LiF/B_4_C peak efficiency ratio 0.54× @ 300 keV/1.20× @ 900 keV	0.0253 eV + 1 MeV; LLD = 300 keV baseline/900 keV
[31]	h-^10^BN epitaxial layer	Semiconductor (h-BN intrinsic)/Planar MSM	enriched ^10^B (isotopically enriched h-^10^BN; purity not stated); ~100 μm; n.r.	normalized/relative (calibrated against certified 30 ± 1% ^6^LiF MSND Domino™ V4, 4 cm^2^): 58.9 ± 2.4%	Moderated ^252^Cf; LLD = highest dark channel
[38]	^10^B_4_C e-beam/Al + PIPS	Semiconductor (PIPS)/Planar	not reported; 2.1 μm; n.r.	not reported	^252^Cf (per title); LLD not reported
[42]	KOH-etched (110)Si + LPCVD 99% ^10^B (1% B_2_H_6_/H_2_, 510 °C)	Semiconductor (trench-filled 3D)/3D trench (continuous p^+^-n junction)	99% ^10^B; 3 μm wide × 40 μm deep, 2 μm Si wall, 2.8 × 2.8 mm^2^ die; n.r.	intrinsic thermal; self-biased 0 V; Cd-shielded background: 16.3 ± 1.6%	^252^Cf/HDPE moderated, 380 n/cm^2^·s @ 8 cm; LLD not reported
[41]	LPCVD enriched ^10^B (99.9%), 800 °C diffusion → continuous p^+^–n junction	Semiconductor (Si diode)/3D honeycomb, DRIE ~2.8 μm wide × ~42 μm deep (AR > 12)	99.9% ^10^B; holes ~2.8 μm wide × ~42 μm deep; n.r.	intrinsic thermal, Maxwellian spectrum, normal incidence, 0 V, 400 keV LLD: 26.1 ± 0.3%	Moderated ^252^Cf (Maxwellian); LLD = 400 keV
[40]	Spin-coated ^10^B_4_C	Semiconductor/3D mechanical-cut trench (nominal 30 μm × 100 μm)	n.r.; n.r.; n.r.	not reported	^241^Am α source (5.486 MeV, α test only, not neutron field)
[24]	SiC + ^6^LiF/elemental ^10^B film/trench/dual layer (Geant4)	Semiconductor/Planar single + dual layer + trench	enriched; 25 μm (^6^LiF planar)/2.5 μm (^10^B planar)/22.5 + 2 μm (dual layer)/100 μm-deep MSND (Tw 15 μm, Tg 5 μm); n.r.	intrinsic (Geant4): 4.3% (^6^LiF planar)/3.9% (^10^B planar)/6.78% (dual); MSND not reported	0.0253 eV thermal; LLD = 300 keV (fixed)

Quantitative comparison of reported thermal-neutron-detection efficiencies across 9 representative conversion-layer studies. Cells marked ‘not reported’ (n.r.) indicate that the corresponding quantity was not reported in the original study. Areal densities given here refer to the converter compound as deposited (e.g., ^6^LiF), whereas the areal-density column of Table 2 reports the isotope loading (^6^Li or ^10^B only); the two quantities are not interchangeable and should not be compared directly. Reported values are not directly comparable because efficiency definition, LLD, and neutron field differ across studies (McGregor & Shultis 2011 [15]); the table is a reference matrix, not a ranking.

## Data Availability

The data presented in this study are available in this article.
